# Reliability of Early Estimates of the Basic Reproduction Number of COVID-19: A Systematic Review and Meta-Analysis

**DOI:** 10.3390/ijerph191811613

**Published:** 2022-09-15

**Authors:** Bibha Dhungel, Md. Shafiur Rahman, Md. Mahfuzur Rahman, Aliza K. C. Bhandari, Phuong Mai Le, Nushrat Alam Biva, Stuart Gilmour

**Affiliations:** 1 Graduate School of Public Health, St. Luke’s International University, Tokyo 104-0045, Japan; 2 Department of Health Policy, National Center for Child Health and Development, Tokyo 157-8535, Japan; 3 Research Centre for Child Mental Development, Hamamatsu University School of Medicine, Hamamatsu 431-3192, Japan; 4 United Graduate School of Child Development, Osaka University, Kanazawa University, Hamamatsu University School of Medicine, Chiba University and University of Fukui, Hamamatsu 431-3192, Japan; 5 Global Public Health Research Foundation, Dhaka 1230, Bangladesh

**Keywords:** basic reproduction number, basic reproductive number, *R*_0_, COVID-19, coronavirus, reliability, pandemic, infectious disease

## Abstract

Objective: This systematic review estimated the pooled $R_{0}$ for early COVID-19 outbreaks and identified the impact of study-related factors such as methods, study location and study period on the estimated $R_{0}$. Methods: We searched electronic databases for human studies published in English between 1 December 2019 and 30 September 2020 with no restriction on country/region. Two investigators independently performed the data extraction of the studies selected for inclusion during full-text screening. The primary outcome, $R_{0}$, was analysed by random-effects meta-analysis using the restricted maximum likelihood method. Results: We identified 26,425 studies through our search and included 151 articles in the systematic review, among which 81 were included in the meta-analysis. The estimates of $R_{0}$ from studies included in the meta-analysis ranged from 0.4 to 12.58. The pooled $R_{0}$ for COVID-19 was estimated to be 2.66 (95% CI, 2.41–2.94). The results showed heterogeneity among studies and strong evidence of a small-study effect. Conclusions: The high heterogeneity in studies makes the use of the $R_{0}$ for basic epidemic planning difficult and presents a huge problem for risk assessment and data synthesis. Consensus on the use of $R_{0}$ for outbreak assessment is needed, and its use for assessing epidemic risk is not recommended.

## 1. Introduction

The World Health Organisation declared a public health emergency of international concern on 30 January 2020 [1], after the outbreak of a novel coronavirus in China that was subsequently named COVID-19. Since that declaration there have been more than 490 million confirmed cases and above 6 million deaths due to COVID-19, affecting more than 180 countries worldwide, and the rate of infection continues to rise [2].

For infectious diseases like COVID-19, the basic reproduction number ($R_{0}$) is essential to understanding the disease transmissibility, preparing preventive measures such as social distancing and lockdowns, and evaluating the effectiveness of policy. The $R_{0}$ is often evaluated early in an emerging infectious disease outbreak to identify the pandemic potential of the disease [3], while the effective reproduction number has been used extensively in some countries to assess the effectiveness of current interventions and the potential to control the epidemic [4].

$R_{0}$ can be estimated using a variety of different methods, based on different forms of data and assumptions about population behaviour and risk. Moreover, early estimates of the $R_{0}$ may be based on limited and highly biased data, and estimates can change over time. Because of the vulnerability of these indices to estimation differences and data quality, they have both been criticised as metrics for assessing either pandemicity or intervention effectiveness [5]. Nonetheless, their use has been widespread in the COVID-19 pandemic, both to make judgments about the effectiveness of highly controversial “herd immunity” strategies [6] and to assess the state of the pandemic at different time points and regions [7]. Many COVID-19 dashboards in many countries report this metric [8,9].

Given these variations in types of reproduction number and methods used to estimate them and the important role this index played in policy assessments in many countries, it is essential to synthesise all existing evidence available to date and summarise the key findings. Previous reviews of the available estimates of $R_{0}$ included a small number of published articles, failed to take into account the different types of effect sizes reported in the study, or did not properly assess publication bias [10,11]. This study aimed to estimate the pooled $R_{0}$ for the COVID-19 outbreak from a full and comprehensive systematic review and meta-analysis of studies published early in the pandemic and identify the impact of study-related factors such as methods, study location and study period on the estimated $R_{0}$.

## 2. Methods

The study was performed according to the protocol registered in PROSPERO (ID = CRD42021279514 [12]) and PRISMA guidelines (Supplementary File S2 and S3).

### 2.1. Search Strategy and Selection Criteria

#### 2.1.1. Database Search

All COVID-19-related studies with title and abstract published between 1 December 2019 and 30 September 2020 were screened. The search was performed in LitCovid, PubMed, MEDLINE, CINAHL, APA PsycInfo, EMBASE, the WHO COVID-19 database, the British Nursing Index, Coronavirus Research Database, Web of Science, CiNii, and the preprint database arXiv. Finally, the reference list of the relevant articles was searched to find additional studies.

#### 2.1.2. Search Strategy

Electronic databases were searched using keywords such as ‘COVID-19’, ‘2019-nCoV’, ‘SARS-CoV-2’, ‘novel coronavirus’, ‘Basic reproduction number’, ‘Basic reproductive rate’ or ‘*R*_0_’, with no restriction on country/region or language but limited to human studies. The search strategies are presented in Supplementary File S1.

#### 2.1.3. Study Selection

Search results were combined, and duplicates were removed. Titles and abstracts were screened using Rayyan QCRI independently by two investigators. When eligibility could not be ascertained, inclusion was decided during full-text screening. Full-text screening was performed by two independent investigators, and disagreements between investigators were resolved by consensus. Original articles reporting reproduction numbers after social interventions, opinion/correspondence, and reviews were also excluded.

#### 2.1.4. Data Extraction and Quality Assessment

Two investigators independently extracted data from the included studies during full-text screening. A standardised data extraction form was prepared (Supplementary File S4) to capture the following information and was pilot-tested. The title of the study, name of the authors, affiliated country of the author, journal, date of publication, study period, study location, model used for estimating $R_{0}$, and the estimated value of $R_{0}$ with 95% confidence interval (CI) or credible interval (CrI) including other intervals were extracted from the selected articles. We used an assessment tool developed by the National Heart, Lung and Blood Institute (NHI) to assess study quality [13]. Any disagreements were resolved by consensus or after discussing with the principal investigator.

### 2.2. Data Analysis

We summarised the findings from the included studies using both narrative synthesis and meta-analysis. A narrative review was used for studies that did not report confidence or credible intervals or other forms of intervals for reproduction numbers, as these could not be included in the meta-analysis. Ranges were converted into confidence intervals using appropriate formulae (Supplementary File S1). Studies with reproduction numbers and estimated confidence intervals were included in the meta-analysis. We first used fixed-effect meta-analysis to obtain the pooled reproduction numbers for studies that estimated multiple reproduction numbers for the same country based on different assumptions and methods. We later utilised this pooled estimate to calculate a summary estimate using a fixed-effect or random-effects meta-analysis based on heterogeneity across studies ($I^{2}$ statistics) [14]. The $I^{2}$, $\tau^{2}$, and Q values were used to examine the extent of heterogeneity between studies. We used the restricted maximum likelihood (REML) method in the case of the random-effects meta-analysis [15]. Log-transformed values of the effect sizes were used in the meta-analysis model, and the results were transformed back to ensure that the pooled effect size was larger than zero (0). Pooled effect sizes, along with a 95% confidence interval, were presented. We assessed the possibility of publication bias through a visual inspection of asymmetry in funnel and Doi plots and through a LFK index to measure asymmetry [16]. When evidence of publication bias was confirmed, we performed the trim-and-fill procedures to account for the possible publication bias [17]. Details on subgroup analysis and sensitivity analysis are presented in Supplementary File S1.

## 3. Results

**Figure 1:**
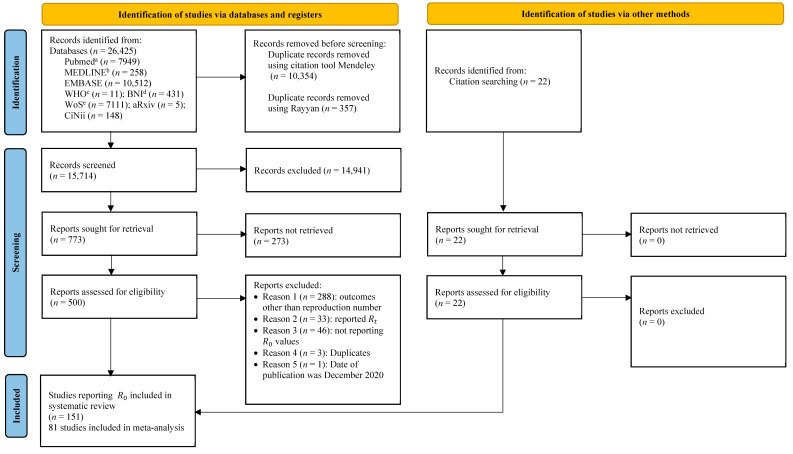
Selection of articles reporting the basic reproduction number of COVID-19 published between 1 December 2019 and 31 September 2020 using a PRISMA flow diagram 2020. Note: ^a^ PubMed, LitCovid, MEDLINE (via PubMed); ^b^ MEDLINE Complete, CINAHL Plus with full text, APA PsychInfo (vis EBSCO host); ^c^ COVID-19 database by the World Health Organization, LILACS (Americas), WPRIM (Western Pacific); ^d^ British Nursing Index, Coronavirus Research Database (via Proquest); ^e^ Web of science.

A schematic representation of the process of selecting articles for this systematic review is shown in Figure 1. This study screened 15,714 articles after removing duplicates from 26,425 identified records. Abstract and title screening resulted in 773 articles with various outcomes. Upon full-text screening, we included 500 articles, out of which 129 articles met the eligibility criteria, and we additionally included 22 articles from the references of the included studies. Finally, 151 articles (Supplementary File S5) estimating $R_{0}$ were included in this study. Seventy-six articles were synthesised narratively, as they did not provide intervals or uncertainty estimates for $R_{0}$. Out of the 76 articles described narratively, six articles that provided interval estimates for some countries were included in the meta-analysis as well. Thus, a total of 81 articles were included in the meta-analysis. The included studies reported reproduction numbers for 73 countries.

**Figure 2:**
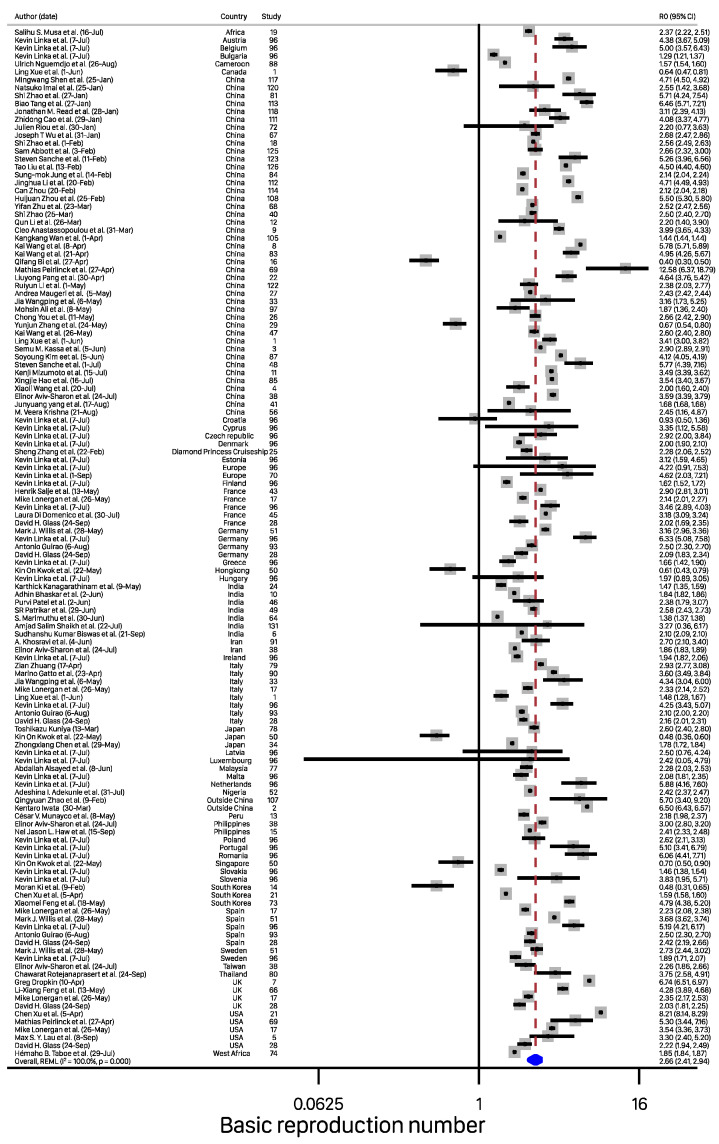
Pooled estimates of basic reproduction number values based on a random effects model. Note: References for the corresponding study numbers can be obtained from Supplementary file S5.

**Figure 3:**
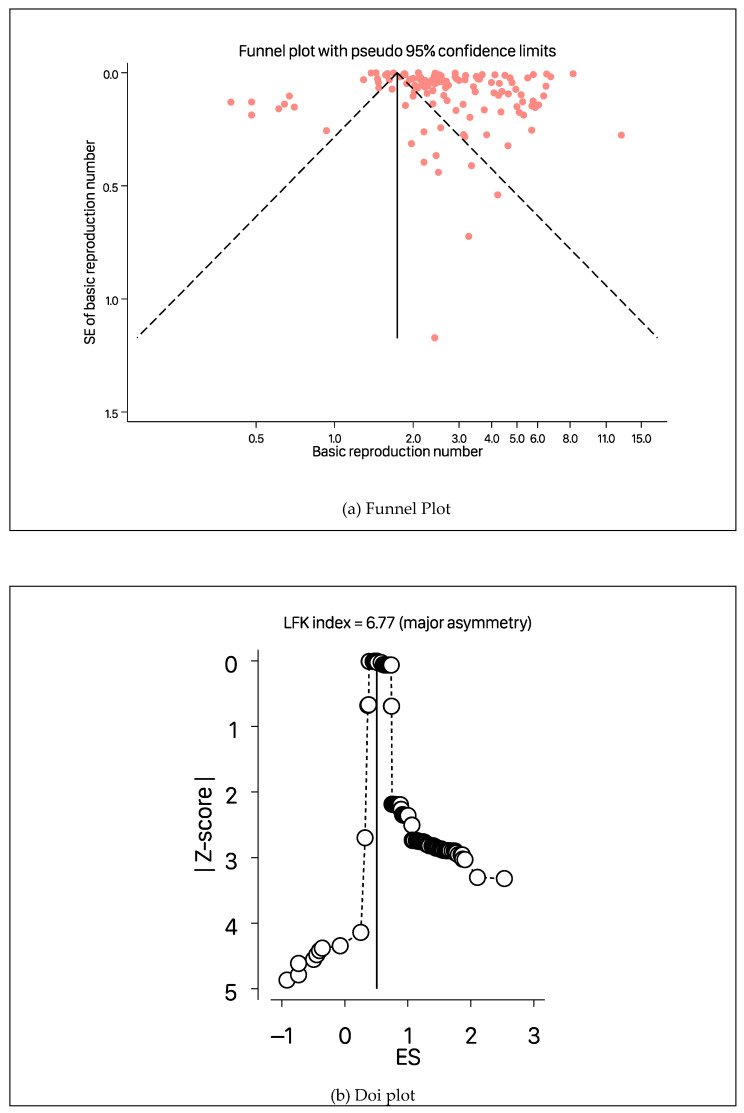

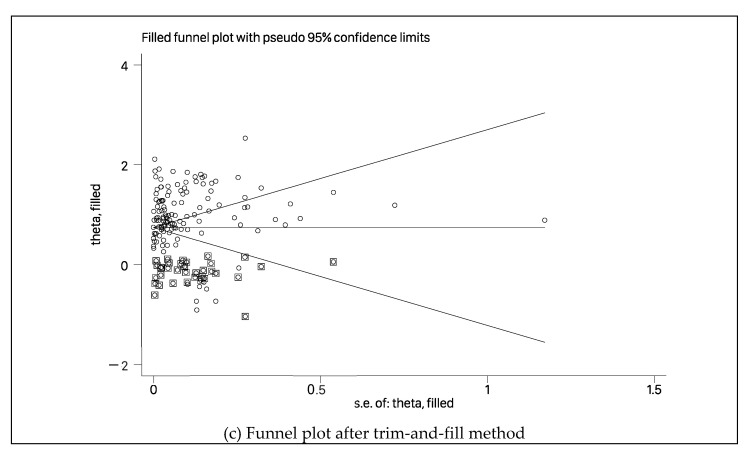
Funnel plot before (**a**) and after (**c**) the trim-and-fill method, and a Doi plot (**b**) for basic reproduction number values based on the random effects model of all studies used to estimate the pooled reproduction number of COVID-19.

The estimates of $R_{0}$ from the studies included in the meta-analysis ranged from 0.4 to 12.58. Details of the studies included in the meta-analysis can be obtained from Table S1 (Supplementary File S1). Figure 2 shows the forest plot with the distribution of $R_{0}$ values by study, with the overall pooled estimate. The pooled $R_{0}$ for COVID-19 was estimated to be 2.66 (95% CI, 2.41–2.94) using a random-effects model. This value suggests that, on average, these studies found that a COVID-19-infected person transmits the infection to around two to three susceptible people. There was heterogeneity among studies ($I^{2}=100\%$, *p*-value from the chi-square test for heterogeneity <0.001, and $\tau^{2}=0.31$). The LFK index of 6.76 (Figure 3b) showed strong evidence of a small-study effect, as indicated by the funnel plot (Figure 3a) and the Doi plot (Figure 3b). The bias-adjusted results from the trim-and-fill method in Figure 3c showed an overall pool estimate of $R_{0}$ of 1.82 (95% CI, 1.74–1.91).

Sub-group analysis is reported in Table 1, and detailed figures are presented in Figures S1–S8 (Supplementary File S1). The pooled estimates for studies using the exponential growth model ($R_{0}$ = 3.06) and compartmental mathematical models ($R_{0}$ = 2.99) were higher than the pooled estimates obtained using the moment-generating function of the Lotka–Euler equation ($R_{0}$ = 2.47), logistic models ($R_{0}$ = 2.60), or other models ($R_{0}$ = 2.24). The overall $R_{0}$ was 2.64, and the pooled estimates were 2.74 for data duration of ≤2 weeks, 2.70 for 2 weeks to 1 month, 2.45 for 1–2 months, and 2.86 for >2 months. This indicated that the estimates prepared in various stages of the epidemic with different periods of data availability were not very different from each other. The pooled estimate of $R_{0}\;$using data collected up to January 2020 was relatively higher ($R_{0}=3.34$) compared to the estimates from subsequent months and was declining until March when more data were available. When COVID-19 started spreading rapidly to different countries, the pooled estimates were highest in studies published in January ($R_{0}=3.87$), while those published in August produced relatively lower estimates, of 2.04. Studies published using data in the USA found higher $R_{0}$ estimates of 4.09 than in India, where the pooled $R_{0}$ was estimated to be 1.91. Similarly, studies from Europe reported higher estimates ($R_{0}=2.74$), while Africa’s pooled $R_{0}$ was 1.94. Studies that reported mean $R_{0}$ had higher pooled estimates, 2.99, compared to studies reporting the median, with pooled estimates of 2.39. In Wuhan, the pooled $R_{0}$ was higher in Wuhan, Hubei (including Wuhan) or overall in China ($R_{0}\sim 3.40$) than outside Hubei in China ($R_{0}=1.50$).

**Figure 4:**
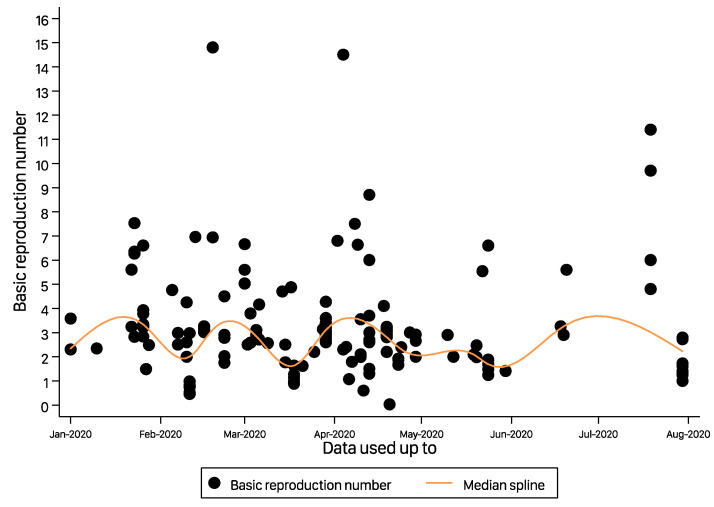
Timeline of the basic reproduction number $\left(R_{0}\right)$ estimates for COVID-19 included in the narrative synthesis.

In sensitivity analysis conducted after excluding studies with $R_{0}$ < 1, the pooled $R_{0}$ was estimated to be 2.92 (95% CI, 2.70–3.16), as shown in Figure S9 (Supplementary File S1). Similarly, an analysis of only good-quality studies estimated a pooled reproduction number ($R_{0}=2.56)$ very close to the overall estimate of 2.66, as shown in Figure S10 (Supplementary File S1). Table S2 (Supplementary File S1) shows that the estimated $R_{0}$ after leave-one-out analysis ranged from 2.63 to 2.70. A study by Bi et al. [18] had the highest influence on the pooled estimate, but it increased the $R_{0}$ by only about 0.4. Figure 4 shows the scatterplot of the $R_{0}$ values of the 76 studies that were narratively ynthesized. A detailed description can be obtained from Table S3 (Supplementary File S1). The estimated reproduction numbers from these studies are in line with the pooled $R_{0}$ estimated from this study, except a few studies that estimated extreme values of $R_{0}$ [19,20,21]. An $R_{0}$ value of 14.8 was estimated in the Diamond Princess Cruise ship using data from 21 January to 19 February 2020 [20].

## 4. Discussion 

This study used meta-analysis to estimate the $R_{0}$ of COVID-19 using a systematic review of articles published between 1 December 2019 and 30 September 2020. We aggregated results published in these studies and synthesised estimates addressing heterogeneity in different studies. When no deliberate intervention was taken for COVID-19, we estimated the $R_{0}$ to be 2.66, with a 95% confidence interval (2.41–2.94), which is slightly higher than the estimates of 1.4 to 2.5 provided by the WHO [22]. Our estimates are similar to the $R_{0}$ of severe acute respiratory syndrome ($R_{0}:$ 2.7; 95% CI: 2.2–3.7) [23] but greater than that of Middle East respiratory syndrome ($R_{0}=0.91$; 95% CI: 0.36–1.44) [24]. Our sub-group analysis found a very wide heterogeneity of estimates in our meta-analysis, with values ranging from 1.91 to 4.09. This shows the vulnerability of $R_{0}$ estimates to choices of modelling methods, data source, location, and timing. Of note, the test for asymmetry in our study indicated the possibility of a small-study effect, meaning that studies with relatively large $R_{0}$ were more likely to be published. If the small-study effect observed in our study was due to publication bias, the true pooled $R_{0}\;$will be 1.82, as estimated by the trim-and-fill method, which is relatively lower than our estimated $R_{0}$ (2.66). However, such a value is inconsistent with the behaviour of the virus in many countries.

Estimating a precise reproduction number is essential for determining the severity and size of any infectious disease and planning interventions to control its spread [25]. However, we found heterogeneity among included studies, which makes the use of the basic reproduction number for basic epidemic planning difficult. The estimated reproduction number of studies included in the meta-analysis ranged from 0.4 to 12.58, orders of magnitude of difference. This heterogeneity was not ameliorated by choice of method, by longer periods of data collection, or by the national origin of the study. Even studies with data collected over periods of greater than 2 months had heterogeneity, and there was heterogeneity independent of the calculation method, nation of origin, or type of central estimate. We included studies from across the world and found wide variation in pooled estimates of $R_{0}$, which ranged from 1.91 in India to 4.09 in the USA. We also found high heterogeneity for estimates within countries, with estimates within single countries varying by orders of magnitude. We found estimated basic reproduction numbers below 1 in 6% of studies (Table 2), which is inconsistent with the rapid spread of the virus during that time period.

This high heterogeneity, which depends heavily on estimation method, data selection, and national characteristics, presents a huge problem for risk assessment and data synthesis. National policymakers, emergency management committees, and the WHO need to be able to make judgments about the pandemic risk of a novel virus. But in order to do so, they must synthesise data on the transmissibility of the virus that is generated with a wide range of different models, has extreme variability, and gives radically different conclusions depending on which study is included in risk assessments and how data is pooled. The reproduction number of COVID-19 in a country is the average of $R_{0}$ in the sub-populations. Thus, even if the overall $R_{0}$ is low or even less than one, it is still necessary to implement strict measures to avert the consequences, as the probability of disease transmission in specific sub-groups of a population may still be high. Failure to take effective and adequate preventive measures may result in serious consequences. Given that we found wide variation in the estimation of $R_{0}$ between studies in the same country or using the same method, the estimation of $R_{0}$ at the sub-national level is unlikely to offer a reliable or useful tool for informing prevention policies.

We estimated that, in the absence of any control measures, a COVID-19-infected individual can transmit the disease to between two and three susceptible people in a naive population. The pooled $R_{0}$ estimate of 2.66 estimated from this study is higher than the early WHO estimate of 1.4–2.5 [22] and indicates a rapid spread of COVID-19. However, many governments and public health decision-makers acted based on lower estimates of $R_{0}$ that are inconsistent with the pooled study estimate found here, with disastrous consequences. For example, the UK Chief Medical Officer, Christ Whitty, announced a “herd immunity” threshold of 60% on national television in March 2020 [32], implying an assumed basic reproduction number of less than 2.5, but by this time only 31% of the published estimates in our study suggested a value in this range. In 2021 the US government set a target of 70% of adults vaccinated, also consistent with an $R_{0}$ value of less than 2.5 [33]. Even though less than 38% of the studies published up to September 2020 found a value in this range. These decisions were inconsistent with the published evidence at that time and failed to take into account the full range of research findings on the infectiousness of the disease. However, with a wide range of published estimates even six months after the novel coronavirus was identified, and no consensus on the correct method for assessing this crucial number, it was very easy for governments to pick values consistent with their political priorities, and impossible to construct a coherent national or global vision for ending coronavirus-related restrictions. The consequences of this have been particularly catastrophic in the USA but have also led to waves of sickness and death in some parts of Europe. The same inherent problems of heterogeneity by method, data source and timing likely also apply to estimates of the infectiousness of subsequent variants of the disease, such as Delta and Omicron, leading to further confusion and inconsistency in decision-making about the pandemic.

The estimation of the reproduction number depends on data sources, environmental factors, serial interval, and model assumptions [34,35]. Ali et al. showed that the serial interval of COVID-19 decreased from 7.8 to 2.6 days between January and February 2020 [35]. This has a large impact on the estimated reproduction numbers, but particularly in the early stages of an outbreak, this serial interval can be under- or over-estimated, and the interpretation of data can lead to significant changes in the estimated reproduction number. However, we also identified studies based on compartmental mathematical modelling which used the next-generation matrix method to estimate $R_{0}$, wherein no information on the serial interval was required and all calculations derived only from observed case numbers. These studies, too, showed very wide variations in $R_{0}\;$estimates, so the problem of inconsistency in estimation is not exclusively due to the serial interval. Our study shows that the competing influence of these factors can lead to a wide range of potential values of $R_{0}$ which make policy decisions difficult. Depending on the study group, data source and method used, the studies we reviewed concluded that the COVID-19 pandemic was disappearing, that the novel coronavirus was no more transmissible than seasonal influenza, that it was a dangerous virus with a pandemic potential twice that of seasonal influenza, or that it was more transmissible than smallpox. Policy responses to an infectious disease of this kind will vary enormously depending on the particular infectiousness regime policymakers believe they are dealing with, but the estimated $R_{0}$ values found within the published literature in 2020 cover such a wide range of regimes as to make policy decisions impossible. This renders this fundamental property of infectious diseases effectively useless for informing policy, and those nations that depended upon this value for determining when to relax restrictions have paid a high price [5,36]. We recommend that the basic reproduction number not be used as policy tools or to inform the public about the current state of pandemics, and that instead, policymakers rely on more precisely calculable measures with public health relevance such as hospital usage, deaths, doubling times, test numbers and positivity rates. Furthermore, the infectious disease modelling and epidemiology community need to develop a consensus on the estimation and reporting of $R_{0}$, how they should be used in emerging infectious disease pandemics, and how they can be understood by laypeople and policymakers. During outbreaks of emerging infectious diseases such as Ebola [37], SARS [23], other novel respiratory viruses [24], and hantaviruses [38], it is common for outbreak analysts to rush early analyses of $R_{0}$ into publication, to inform national and global policymakers of the pandemic risk associated with the outbreak. This systematic review shows that these estimates are highly sensitive to assumptions, modelling methods and data and cannot be relied upon to provide meaningful or comparable information about the nature of emerging pandemics. We therefore recommend that, in preparing for the next global pandemic or public health emergency of international concern, the WHO convene a working group to establish clear guidelines for the calculation, reporting and use of reproduction numbers, as well as information for policymakers, and the global health community should consider establishing a single, globally agreed research group tasked with assessing outbreaks within a commonly agreed framework endorsed by the WHO. Models may underestimate or overestimate reproduction numbers depending on whether unobserved and asymptomatic cases are considered. Although some of the papers reviewed incorporated this information, we did not incorporate this information in the current study. A follow-up study with detailed assessment of study quality, methods and estimation tools is required to assess how the estimates of reproduction numbers vary among studies that included or did not include unobserved cases, or by other details of the modelling process (such as estimation methods, data sources and calibration or validation methods used). A great deal of additional theoretical and quality assessment is needed to understand which studies are best placed to contribute to scientific knowledge about pandemic risk and to what extent data sources or modelling methods can be misleading or inaccurate.

## 5. Conclusions

The global understanding of infectious disease outbreaks remains weak, and the novel coronavirus pandemic is the first rapidly spreading global pandemic since the 2018 Spanish influenza pandemic, which occurred at a time when sophisticated data analysis and disease modelling were not available. This study shows that there is still much theoretical and practical work to be done before we can properly understand the dynamics of emerging infectious diseases and that $R_{0}$ offers a highly variable measure of pandemic risk, subject to much uncertainty and vulnerable to the influence of modelling assumptions, data quality and data timeliness. Although infectious disease models and composite emergent indicators such as $R_{0}$ offer a tempting tool to simplify the understanding of pandemics, they do not offer the clarity and precision needed to make decisions in a global pandemic. Until the epidemiological community has a clearer understanding of how to use these measures, they should be deprecated in favour of basic public health principles that offer a clear, simple framework for pandemic response. Until we have a clearer understanding of and consensus on how to use infectious disease models for pandemic response, we cannot hope to prepare for the next pandemic.

## Figures and Tables

**Table 1:** Sub-group analysis for basic reproduction number ($R_{0}$ ) based on a random effects model.

Characteristics	Number of Reporting	*R*_0_ (95% CI)	*p* Value
			Heterogeneity
Method considered	(*n* = 161)		
Exponential growth model	20	3.06 (2.32–4.03)	<0.001
Moment generating function of the Lotka–Euler equation	6	2.47 (2.13–2.86)	<0.001
Compartmental model	87	2.99 (2.67–3.35)	<0.001
Logistic model	4	2.60 (1.94–3.48)	<0.001
Others	44	2.24 (1.87–2.69)	<0.001
Duration of data	(*n* = 127)		
≤2 weeks	15	2.74 (2.26–3.31)	<0.001
2 weeks–1 month	28	2.70 (2.11–3.46)	<0.001
1–2 months	53	2.45 (2.06–2.91)	<0.001
>2 months	31	2.86 (2.47–3.32)	<0.001
Last month of data	(*n* = 128)		
January	25	3.34 (2.89–3.87)	<0.001
February	14	2.23 (1.40–3.56)	<0.001
March	30	2.18 (1.73–2.76)	<0.001
April	13	2.72 (1.99–3.71)	<0.001
May	12	2.69 (2.40–3.01)	<0.001
June	30	2.80 (2.31–3.39)	<0.001
July	4	2.60 (1.94–3.48)	<0.001
Month of publication	(*n* = 130)		
January	8	3.87 (2.97–5.03)	<0.001
February	11	2.90 (1.92–4.37)	<0.001
March	6	3.18 (2.28–4.45)	<0.001
April	11	3.37 (1.93–5.89)	<0.001
May	26	2.22 (1.74–2.85)	<0.001
June	11	2.12 (1.58–2.86)	<0.001
July	40	2.83 (2.43–3.28)	<0.001
August	6	2.04 (1.70–2.45)	<0.001
September	8	2.27 (2.12–2.43)	<0.001
Country	(*n* = 130)		
China	43	3.02 (2.55–3.59)	<0.001
Other	49	2.24 (1.87–2.68)	<0.001
USA	5	4.09 (2.60–6.43)	<0.001
Italy	8	2.69 (2.08–3.48)	<0.001
India	7	1.91 (1.56–2.33)	<0.001
France	5	2.68 (2.18–3.29)	<0.001
UK	4	3.43 (1.99–5.91)	<0.001
Spain	5	3.02 (2.22–4.09)	<0.001
Germany	3	3.18 (1.99–5.08)	<0.001
Continent	(*n* = 126)		
Asia	66	2.54 (2.18–2.96)	<0.001
Europe	50	2.78 (2.46–3.15)	<0.001
North America	8	2.74 (1.62–4.64)	0.002
Africa	2	1.94 (1.27–2.98)	<0.001
Type of central estimate	(*n* = 130)		
Mean	34	2.99 (2.43–3.68)	<0.001
Median	13	2.39 (1.91–2.98)	<0.001
Other	83	2.58 (2.28–2.92)	<0.001
Location in China	(*n* = 43)		
Wuhan	8	3.40 (2.61–4.44)	<0.001
Hubei including Wuhan	2	3.39 (2.48–4.64)	<0.001
Outside Hubei in China	6	1.50 (0.76–2.96)	<0.001
China overall	27	3.39 (2.84–4.04)	<0.001

**Table 2:** Basic reproduction number ($R_{0}$) of various infectious diseases with proportion of studies reporting $R_{0}\;$ within the given threshold.

Reproduction Number Threshold	Number (%)	Cumulative Number (%)	Mean $R_{0}$	Range
Epidemic containment ($R_{0}$ < 1)	21 (6.2%)	21 (6.2%)	0.69	0.03–0.99
Influenza (1 ≤ $R_{0}$ < 1.5) [26]	19 (5.6%)	40 (11.8%)	1.33	1.00–1.49
SARS-CoV (1.5 ≤ $R_{0}$ < 4) [23]	231 (68.3%)	271 (80.2%)	2.61	1.50–3.99
HIV (4 ≤ $R_{0}$ < 5) [27]	26 (7.7%)	297 (87.9%)	4.43	4.02–4.95
Smallpox (5 ≤ $R_{0}$ < 6) [28]	16 (4.7%)	313 (92.6%)	5.47	5.00–5.88
Rubella/Polio (6 ≤ $R_{0}$ < 7) [29]	16 (4.7%)	329 (97.3%)	6.49	6–6.96
Chickenpox (7 ≤ $R_{0}$ < 12) [30]	6 (1.8%)	335 (99.1%)	8.84	7.50–11.40
Measles (12 ≤ $R_{0}$ < 18) [31]	3 (0.9%)	338 (100%)	13.96	12.58–14.80

## Data Availability

All data used in this study have been provided in the Supplementary File S1 (Tables S1 and S3).

## Supplementary Materials

The following supporting information can be downloaded at: https://www.mdpi.com/article/10.3390/ijerph191811613/s1, Supplementary data are available online. Supplementary File S1: Conversion of effect sizes, Search strategy and selection criteria, Search strategies, Statistical analysis: sub-group and sensitivity analysis; Figure S1: Sub-group analysis of basic reproduction number by method; Figure S2: Sub-group analysis of basic reproduction number by duration of data; Figure S3: Sub-group analysis of basic reproduction number by last month of data; Figure S4: Sub-group analysis of basic reproduction number by month of publication; Figure S5: Sub-group analysis of basic reproduction number by country; Figure S6: Sub-group analysis of basic reproduction number by type of central estimate; Figure S7: Sub-group analysis of basic reproduction number by location in China; Figure S8: Sub-group analysis of basic reproduction number by continent; Figure S9: Sensitivity analysis of basic reproduction number excluding studies with $R_{0}$ ≤ 1; Figure S10: Sensitivity analysis of basic reproduction number excluding fair or low-quality studies; Table S1: Descriptive characteristics including quality assessment of studies with basic reproduction number included in meta-analysis; Table S2. Result of influence analysis for basic reproduction number; Table S3: Narrative synthesis including quality assessment of the studies with basic reproduction number included in the review; Supplementary File S2: Protocol registered in PRESPERO; Supplementary File S3: PRISMA 2020 Checklist; Supplementary File S4: Data extraction form; Supplementary File S5. References included in the systematic review and meta-analysis (cited in Figure 2).
