# Supplementary material for: Reliability of Early Estimates of the Basic Reproduction Number of COVID-19: A Systematic Review and Meta-Analysis
Source: Int J Environ Res Public Health. 2022 Sep 15;19(18):11613. doi: 10.3390/ijerph191811613 (PMC9517346; doi:10.3390/ijerph191811613)
Supplement: Supplementary file 1 [file ijerph-19-11613-s001.zip › Supplementary file S1.pdf]

Reliability of Early Estimates of Basic Reproduction Number of COVID-19: A Systematic Review and Meta-Analysis

Bibha Dhungel[1,2], Md. Shafiur Rahman[3,4], Md. Mahfuzur Rahman[5], Aliza KC Bhandari[1,2], Phuong Mai Le[1], Nushrat Alam Biva[1], Stuart Gilmour[1]

[1]Graduate School of Public Health, St. Luke's International University, Tokyo, Japan
[2]Department of Health Policy, National Center for Child Health and Development, Tokyo, Japan
[3]Research Centre for Child Mental Development, Hamamatsu University School of Medicine, Hamamatsu, Japan
[4]United Graduate School of Child Development, Osaka University, Kanazawa University, Hamamatsu University School of Medicine, Chiba University and University of Fukui, Hamamatsu, Japan

[5]Global Public Health Resarch Foundation, Dhaka, Bangladesh

Corresponding author
Bibha Dhungel
Graduate School of Public Health, St. Luke's International University, Tsukiji 3-6-2, Tokyo 104-0045, Japan
18mp201@slcn.ac.jp

Table of Contents

Conversion of effect sizes 3
Search strategy and selection criteria 4
Search strategies 4

PubMed, LitCovid, MEDLINE (via PubMed) 4
MEDLINE Complete, CINAHL Plus with full text, APA PsychInfo (vis EBSCO host) 4
EMBASE 5
COVID-19 database by the World Health Organization, LILACS (Americas), WPRIM (Western Pacific) 5
British Nursing Index, Coronavirus Research Database (via Proquest) 5
Web of science 6
aRxiv 6
CiNii 6

Statistical analysis: sub-group and sensitivity analysis 7
Supplementary figures 8

Figure S1. Sub-group analysis of basic reproduction number by method 8
Figure S2. Sub-group analysis of basic reproduction number by duration of data 9
Figure S3. Sub-group analysis of basic reproduction number by last month of data 10
Figure S4. Sub-group analysis of basic reproduction number by month of publication 11
Figure S5. Sub-group analysis of basic reproduction number by country 12
Figure S6. Sub-group analysis of basic reproduction number by type of central estimate 13
Figure S7. Sub-group analysis of basic reproduction number by location in China 14
Figure S8. Sub-group analysis of basic reproduction number by continent 15
Figure S9. Sensitivity analysis of basic reproduction number excluding studies with $R0 \leq 1$ 16
Figure S10. Sensitivity analysis of basic reproduction number excluding fair or low-quality studies 17

Supplementary tables 18

Table S1. Descriptive characteristics including quality assessment of studies with basic reproduction number included in meta-analysis 18
Table S2. Result of influence analysis for basic reproduction number 19
Table S3. Narrative synthesis including quality assessment of the studies with basic reproduction number included in the review 22

Conversion of effect sizes

Studies reported reproduction numbers with various interval estimates such as 90 or 95% confidence interval, 95% credible interval, 90% high density interval, mean±standard error, interquartile range, upper and lower quartile, maximum & minimum, and range. Standard error was calculated using appropriate formulae as below, which was then converted to 95% confidence interval to use comparable effect sizes in the meta-analysis.

Step 1: Calculating Standard Error

i. For 90 % Confidence or credible or high-density interval

$$SE = \frac{UCI - LCI}{2 * invnormal(0.95)}$$

ii. For 95% Confidence or credible interval

$$SE = \frac{UCI - LCI}{2 * invormal(0.975)}$$

iii. One study has credible interval but no mean value. Calculated mean value as:

$$SE = \frac{UCI - LCI}{2 * invnormal(0.95)}$$

$$R0 = UCI - (invnormal(0.95) * SE)$$

iv. Two studies have SD but no interval. Calculated SE as:

$$SE = \frac{SD}{\sqrt{n}}$$

v. Interquartile range / lower and upper quartile: 3 studies

$$SD = \frac{UCI - LCI}{1.35}$$

$$SE = \frac{SD}{\sqrt{n}}$$

vi. Studies with Lower/Upper values or Minimum/Maximum or range

$mean = \frac{(LCI + 2*Median + UCI)}{4}$

$$SD = \sqrt{\left(\frac{1}{12} * \left(\frac{(LCI - 2 * Median + UCI)^2}{4} + (UCI - LCI)^2\right)\right)}$$

$SE = \frac{SD}{\sqrt{n}}$ if n if given

$SE = SD$ if n is not given

vii. If R_0± XX, even if it is not mentioned whether the value after ± is SE or SD, it is assumed to be SE

viii. If R_0 ± SD, and sample size not available. SE = SD

Step 2: Calculationg 95% confidence interval

After calculating standard error for all observations, 95% confidence interval (CI) of R_0 was calculated as:

95% Lower CI $= R_0 - 1.96 * SE$

95% Upper CI $= R_0 + 1.96 * SE$

Search strategies

PubMed, LitCovid, MEDLINE (via PubMed)

	Keywords
#1	(((coronavirus*[Title/Abstract]) OR (coronavirus infection[Title/Abstract]) OR (coronavirus disease[Title/Abstract]) OR (coronavirus disease 2019[Title/Abstract]) OR (coronavirus disease-19[Title/Abstract]) OR (coronavirus disease 19[Title/Abstract]) OR (covid19[Title/Abstract]) OR (covid 19[Title/Abstract]) OR (covid-19[Title/Abstract]) OR (covid-19 pandemic[Title/Abstract]) OR (covid 19 pandemic[Title/Abstract]) OR (covid-19 virus infection[Title/Abstract]) OR (covid 19 virus infection[Title/Abstract]) OR (covid-19 virus disease[Title/Abstract]) OR (covid 2019[Title/Abstract]) OR (sars-cov-2[Title/Abstract]) OR (sars cov 2[Title/Abstract]) OR (severe acute respiratory syndrome coronavirus 2[Title/Abstract]) OR (severe acute respiratory syndrome coronavirus 2[Supplementary Concept]) OR (novel coronavirus 2019[Title/Abstract]) OR (2019 novel coronavirus disease[Title/Abstract]) OR (2019 novel coronavirus infection[Title/Abstract]) OR (2019-nCoV infection[Title/Abstract]) OR (2019-nCoV[Title/Abstract]) OR (2019nCov[Title/Abstract]) OR (2019 nCov[Title/Abstract]))
#2	((Basic Reproduction Number[Title/Abstract]) OR (Basic reproductive number[Title/Abstract]) OR (Morbidity[Title/Abstract]) OR (Mortality[Title/Abstract]) OR (Mortality rate[Title/Abstract]) OR (Case fatality rate[Title/Abstract]) OR (Incidence[Title/Abstract]) OR (Prevalence[Title/Abstract]) OR (Serial interval[Title/Abstract]) OR (Attack rate[Title/Abstract]) OR (Rate change[Title/Abstract]) OR (Percentage change[Title/Abstract]) OR (Percent change[Title/Abstract]) OR (Reduction rate[Title/Abstract]) OR (Absolute change[Title/Abstract]) OR (Reduction[Title/Abstract]) OR (Reduc*[Title/Abstract]) OR (Death[Title/Abstract]) OR (Excess death[Title/Abstract]) OR (Asymptomatic[Title/Abstract]) OR (Asymptomatic Infection[Title/Abstract]) OR (Asymptomatic Disease[Title/Abstract]) OR (Asymptomatic covid-19[Title/Abstract]) OR (Infection rate[Title/Abstract]) OR (Misreport*[Title/Abstract]) OR (Misreporting rate[Title/Abstract]) OR (Forecast*[Title/Abstract]) OR (Forecasting[Title/Abstract]) OR (Population Forecast[Title/Abstract]) OR (Prediction[Title/Abstract]) OR (trends[Title/Abstract]) OR (trend*[Title/Abstract]) OR (trend analysis[Title/Abstract]) OR (Lockdown[Title/Abstract]) OR (Lock down[Title/Abstract]) OR (travel ban[Title/Abstract]) OR (travel restriction[Title/Abstract]) OR (travel restrictions[Title/Abstract]) OR (quarantine[Title/Abstract]) OR (shut down[Title/Abstract]) OR (shutdown[Title/Abstract])))
#3	#1 AND #2 (human, 2019/12/1-2020/9/30)<br>Search performed on: 15th October 2020<br>Total: 7,949

MEDLINE Complete, CINAHL Plus with full text, APA PsychInfo (vis EBSCO host)

	Keywords
S1	AB coronavirus* OR AB "coronavirus infection" OR AB "coronavirus disease" OR AB "coronavirus disease 2019" OR AB "coronavirus disease-19" OR AB "coronavirus disease 19" OR AB covid19 OR AB "covid 19" OR AB "covid-19" OR AB "covid-19 pandemic" OR AB 2covid 19 pandemic" OR AB "covid-19 virus infection"
S2	AB "covid 19 virus infection" OR AB "covid-19 virus disease" OR AB "covid 2019" OR AB "sars-cov-2" OR AB "sars cov 2" OR AB "severe acute respiratory syndrome coronavirus 2" OR AB "novel coronavirus 2019" OR AB "2019 novel coronavirus disease" OR AB "2019 novel coronavirus infection" OR AB "2019-nCoV infection" OR AB "2019-nCoV" OR AB 2019nCov
S3	AB 2019 nCov
S4	AB S1 OR S2 OR S3
S5	AB "Basic Reproduction Number" OR AB "Basic reproductive number" OR AB Morbidity OR AB Mortality OR AB "Mortality rate" OR AB "Case fatality rate" OR AB Incidence OR AB Prevalence OR AB "Serial interval" OR AB "Attack rate" OR AB "Rate change" OR AB "Percentage change"
S6	AB "Percent change" OR AB "Reduction rate" OR AB "Absolute change" OR AB Reduction OR AB Reduc* OR AB Death OR AB "Excess death" OR AB Asymptomatic OR AB "Asymptomatic Infection2 OR AB "Asymptomatic Disease" OR AB "Asymptomatic covid 19" OR AB "Infection rate"
S7	AB S5 OR S6
S8	AB S4 AND S7
S9	AB S4 AND S7<br>Limiters - Date of Publication: 20190101-20200930<br>Narrow by Population: - human<br>Search performed on: 16th October 2020<br>Total: 258

EMBASE

	Keywords
#1	coronavirus*:ab,ti OR 'coronavirus infection':ab,ti OR 'coronavirus disease':ab,ti OR 'coronavirus disease 2019':ab,ti OR 'coronavirus disease-19':ab,ti OR 'coronavirus disease 19':ab,ti OR covid19:ab,ti OR 'covid 19':ab,ti OR 'covid-19 pandemic':ab,ti OR 'covid 19 pandemic':ab,ti OR 'covid-19 virus infection':ab,ti OR 'covid 19 virus infection':ab,ti OR 'covid-19 virus disease':ab,ti OR 'covid 2019':ab,ti OR 'sars-cov-2':ab,ti OR 'sars cov 2':ab,ti OR 'severe acute respiratory syndrome coronavirus 2':ab,ti OR 'novel coronavirus 2019':ab,ti OR '2019 novel coronavirus disease':ab,ti OR '2019 novel coronavirus infection':ab,ti OR '2019-ncov infection':ab,ti OR '2019-ncov':ab,ti OR '2019ncov':ab,ti OR '2019 ncov':ab,ti
#2	'basic reproduction number':ab,ti OR 'basic reproductive number':ab,ti OR morbidity:ab,ti OR mortality:ab,ti OR 'mortality rate':ab,ti OR 'case fatality rate':ab,ti OR incidence:ab,ti OR prevalence:ab,ti OR 'serial interval':ab,ti OR 'attack rate':ab,ti OR 'rate change':ab,ti OR 'percentage change':ab,ti OR 'percent change':ab,ti OR 'reduction rate':ab,ti OR 'absolute change':ab,ti OR reduction:ab,ti OR reduc*:ab,ti OR death:ab,ti OR 'excess death':ab,ti OR asymptomatic:ab,ti OR 'asymptomatic infection':ab,ti OR 'asymptomatic disease':ab,ti OR 'asymptomatic covid 19':ab,ti OR 'infection rate':ab,ti OR misreport*:ab,ti OR 'misreporting rate':ab,ti OR forecast*:ab,ti OR forecasting:ab,ti OR 'population forecast':ab,ti OR prediction:ab,ti OR trends:ab,ti OR trend*:ab,ti OR 'trend analysis':ab,ti OR lockdown:ab,ti OR 'lock down':ab,ti OR 'travel ban':ab,ti OR 'travel restriction':ab,ti OR 'travel restrictions':ab,ti OR quarantine:ab,ti OR 'shut down':ab,ti OR shutdown:ab,ti
#3	#1 AND #2
#4	#1 AND #2 AND [humans]/lim AND [embase]/lim AND [2019-2020]/py
	Search performed on: 16th October 2020
	Total: 10,512

COVID-19 database by the World Health Organization, LILACS (Americas), WPRIM (Western Pacific)

	Keywords
A	(tw:(coronavirus*)) OR (tw:("coronavirus infection")) OR (tw:("coronavirus disease")) OR (tw:("coronavirus disease 2019")) OR (tw:("coronavirus disease-19 ")) OR (tw:("coronavirus disease 19")) OR (tw:("covid19")) OR (tw:("covid 19")) OR (tw:("covid-19 pandemic")) OR (tw:("covid 19 pandemic")) OR (tw:("covid-19 virus infection")) OR (tw:("covid 19 virus infection")) OR (tw:("covid-19 virus disease")) OR (tw:("covid 2019")) OR (tw:("sars-cov-2")) OR (tw:("sars cov 2")) OR (tw:("severe acute respiratory syndrome coronavirus 2")) OR (tw:("novel coronavirus 2019")) OR (tw:("2019 novel coronavirus disease")) OR (tw:("2019 novel coronavirus infection")) OR (tw:("2019-nCoV infection")) OR (tw:("2019-nCoV")) OR (tw:(2019nCov)) OR (tw:("2019 nCov"))
B	(tw:("Basic Reproduction Number")) OR (tw:("Basic reproductive number")) OR (tw:(Morbidity)) OR (tw:(Mortality)) OR (tw:("Mortality rate")) OR (tw:("Case fatality rate")) OR (tw:(Incidence)) OR (tw:(Prevalence)) OR (tw:("Serial interval")) OR (tw:("Attack rate")) OR (tw:("Rate change")) OR (tw:("Percentage change")) OR (tw:("Percent change")) OR (tw:("Reduction rate")) OR (tw:("Absolute change")) OR (tw:("Reduction")) OR (tw:("Reduc*")) OR (tw:("Death")) OR (tw:("Excess death")) OR (tw:(Asymptomatic)) OR (tw:("Asymptomatic Infection")) OR (tw:("Asymptomatic Disease")) OR (tw:("Asymptomatic covid 19")) OR (tw:("Infection rate")) OR (tw:("Misreport*")) OR (tw:("Misreporting rate")) OR (tw:(Forecast*)) OR (tw:(Forecasting)) OR (tw:(Population Forecast)) OR (tw:(Prediction)) OR (tw:(trends)) OR (tw:(trend*)) OR (tw:("trend analysis")) OR (tw:(Lockdown)) OR (tw:("Lock down")) OR (tw:("travel ban")) OR (tw:("travel restriction")) OR (tw:("travel restrictions")) OR (tw:(quarantine)) OR (tw:("shut down")) OR (tw:(shutdown))
	A AND B
	Search performed on: 18th October 2020
	Total: 11

British Nursing Index, Coronavirus Research Database (via Proquest)

	Keywords
A	(ab(coronavirus*) OR ab("coronavirus infection") OR ab("coronavirus disease") OR ab("coronavirus disease 2019") OR ab("coronavirus disease-19 ") OR ab("coronavirus disease 19") OR ab("covid19") OR ab("covid 19") OR ab("covid-19") OR ab("covid-19 pandemic") OR ab("covid 19 pandemic") OR ab("covid-19 virus infection") OR ab("covid 19 virus infection") OR ab("covid-19 virus disease") OR ab("covid 2019") OR ab("sars-cov-2") OR ab("sars cov 2") OR ab("severe acute respiratory syndrome coronavirus 2") OR ab("novel coronavirus 2019") OR ab("2019 novel coronavirus disease"))
B	(ab("basic reproduction number") OR ab("basic reproductive rate") OR ab("morbidity") OR ab("Mortality") OR ab("mortality rate") OR ab("case fatality rate") OR ab("incidence") OR ab("prevalence") OR ab("Serial interval") OR ab("Attack rate") OR ab("rate change") OR ab("Percentage change") OR ab("Percent change") OR ab("Reduction rate") OR ab("Absolute change") OR ab(Reduction") OR ab(Reduc*") OR ab("Death") OR ab("Excess death") OR ab(Asymptomatic) OR ab("asymptomatic infections") OR ab("asymptomatic diseases") OR ab("Asymptomatic covid-19") OR ab("infection rate") OR Misreport* OR ab("Misreporting rate") OR ab(Forecast*) OR ab("Forecasting") OR ab("population forecast") OR ab(Prediction) OR ab("trends") OR ab(trend*) OR ab("trends analysis") OR ab(Lockdown) OR ab("Lock down") OR ab("travel ban") OR ab("travel restriction") OR ab("travel restrictions") OR ab(quarantine) OR ab("shut down") OR ab(shutdown))
	A and B (2019-12-01 - 2020-09-30)(British Nursing Index)
	Search performed on: 16th October 2020
	Total: 431

Web of science

	Keywords
#1	(TS=(coronavirus*) OR TS=("coronavirus infection") OR TS=("coronavirus disease") OR TS=("coronavirus disease 2019") OR TS= ("coronavirus disease-19 ") OR TS= ("coronavirus disease 19") OR TS= ("covid19") OR TS= ("covid 19") OR TS= ("covid-19") OR TS= ("covid-19 pandemic") OR TS= ("covid 19 pandemic") OR TS= ("covid-19 virus infection") OR TS= ("covid 19 virus infection") OR TS= ("covid-19 virus disease") OR TS= ("covid 2019") OR TS= ("sars-cov-2") OR TS= ("sars cov 2") OR TS=("severe acute respiratory syndrome coronavirus 2") OR TS= ("novel coronavirus 2019") OR TS= ("2019 novel coronavirus disease") OR TS= ("2019 novel coronavirus infection") OR TS= ("2019-nCoV infection") OR TS= ("2019-nCoV") OR TS= ("2019nCov") OR TS=("2019 nCov"))
#2	(TS= ("Basic Reproduction Number") OR TS=("Basic reproductive number") OR TS= ("Morbidity") OR TS=("Mortality") OR TS= ("Mortality rate") OR TS= ("Case fatality rate") OR TS= ("Incidence") OR TS=("Prevalence") OR TS= ("Serial interval") OR TS= ("Attack rate") OR TS= ("Rate change") OR TS=("Percentage change") OR TS=("Percent change") OR TS=("Reduction rate") OR TS=("Absolute change") OR TS=("Reduction") OR TS= ("Reduc*") OR TS= ("Death") OR TS=("Excess death") OR TS=(Asymptomatic) OR TS=("Asymptomatic Infection") OR TS=("Asymptomatic Disease") OR TS=("Asymptomatic covid 19") OR TS=("Infection rate") OR TS=("Misreport*") OR TS=("Misreporting rate") OR TS=(Forecast*) OR TS= (Forecasting) OR TS= ("Population Forecast") OR TS= ("Prediction") OR TS=("trends") OR TS= (trend*) OR TS= ("trend analysis") OR TS=(Lockdown) OR TS= ("Lock down") OR TS= ("travel ban") OR TS=("travel restriction") OR TS=("travel restrictions") OR TS=("quarantine") OR TS= ("shut down") OR TS= ("shutdown"))
#3	#1 AND #2 (2019 to 2020) (Refined by: DOCUMENT TYPES: (ARTICLE OR EARLY ACCESS))<br>Search performed on: 16th October 2020<br>Total: 7,111

aRxiv

	Keywords
A	"coronavirus*" OR "coronavirus infection" OR "covid 19" OR "coronavirus disease" OR "covid19" OR "covid 2019" OR "sars-cov-2"
B	“Basic Reproduction Number” OR “Basic reproductive number” OR “Morbidity” OR “Mortality” OR “Mortality rate” OR “Case fatality rate” OR “Incidence” OR “Prevalence” OR “Serial interval“ OR “Attack rate” OR “Rate change” OR “Percentage change” OR “Percent change” OR “Reduction rate” OR “Absolute change” OR “Reduction” OR “Reduc*” OR “Death” OR “Excess death” OR “Asymptomatic” OR “Asymptomatic Infection” OR “Asymptomatic Disease” OR “Asymptomatic covid-19” OR “Infection rate” OR “Misreport*” OR “Misreporting rate” OR “Forecast*” OR “Forecasting” OR “Population Forecast” OR “Prediction” OR “trends” OR “trend*” OR “trend analysis” OR “Lockdown” OR “Lock down” OR “travel ban” OR “travel restriction” OR “travel restrictions” OR “quarantine” OR “shut down” OR “shutdown”
	A AND B<br>Search performed on: 16th October 2020<br>Total: 5

CiNii

	Keywords
A	(“coronavirus*” OR “coronavirus infection” OR “coronavirus disease” OR “coronavirus disease 2019” OR “coronavirus disease-19” OR “coronavirus disease 19” OR “covid19” OR “covid 19” OR “covid-19” OR “covid-19 pandemic” OR “covid 19 pandemic” OR “covid-19 virus infection” OR “covid 19 virus infection” OR “covid-19 virus disease” OR “covid 2019” OR “sars-cov-2” OR “sars cov 2” OR “severe acute respiratory syndrome coronavirus 2” OR “novel coronavirus 2019” OR “2019 novel coronavirus disease” OR “2019 novel coronavirus infection” OR “2019-nCoV infection” OR “2019-nCoV” OR “2019nCov” OR “2019 nCov”)
B	(“Basic Reproduction Number” OR “Basic reproductive number” OR “Morbidity” OR “Mortality” OR “Mortality rate” OR “Case fatality rate” OR “Incidence” OR “Prevalence” OR “Serial interval“ OR “Attack rate” OR “Rate change” OR “Percentage change” OR “Percent change” OR “Reduction rate” OR “Absolute change” OR “Reduction” OR “Reduc*” OR “Death” OR “Excess death” OR “Asymptomatic” OR “Asymptomatic Infection” OR “Asymptomatic Disease” OR “Asymptomatic covid-19” OR “Infection rate” OR “Misreport*” OR “Misreporting rate” OR “Forecast*” OR “Forecasting” OR “Population Forecast” OR “Prediction” OR “trends” OR “trend*” OR “trend analysis” OR “Lockdown” OR “Lock down” OR “travel ban” OR “travel restriction” OR “travel restrictions” OR “quarantine” OR “shut down” OR “shutdown”)
	A AND B<br>Search performed on: 16th October 2020<br>Total: 148

Statistical analysis: sub-group and sensitivity analysis

We also conducted a sub-group analysis by country, continent, study duration and method, whether mean or median was reported for reproduction number, month of publication, and whether the study was conducted in Wuhan, Hubei including Wuhan or outside Hubei in China. Influence analysis was performed using leave-one-out analysis to detect which study influenced the pooled estimate of the meta-analysis most. Sensitivity analysis was conducted by leaving the most influential studies from the analysis, excluding studies with $R_0<1$ considering the increasing number of cases with the spread of the COVID-19 virus and excluding fair or low-quality studies. Version 14.2 of Stata software (Stata Corp, College Station, Texas, USA) was used to perform the analyses.

Supplementary figures

Figure S1. Sub-group analysis of basic reproduction number by method

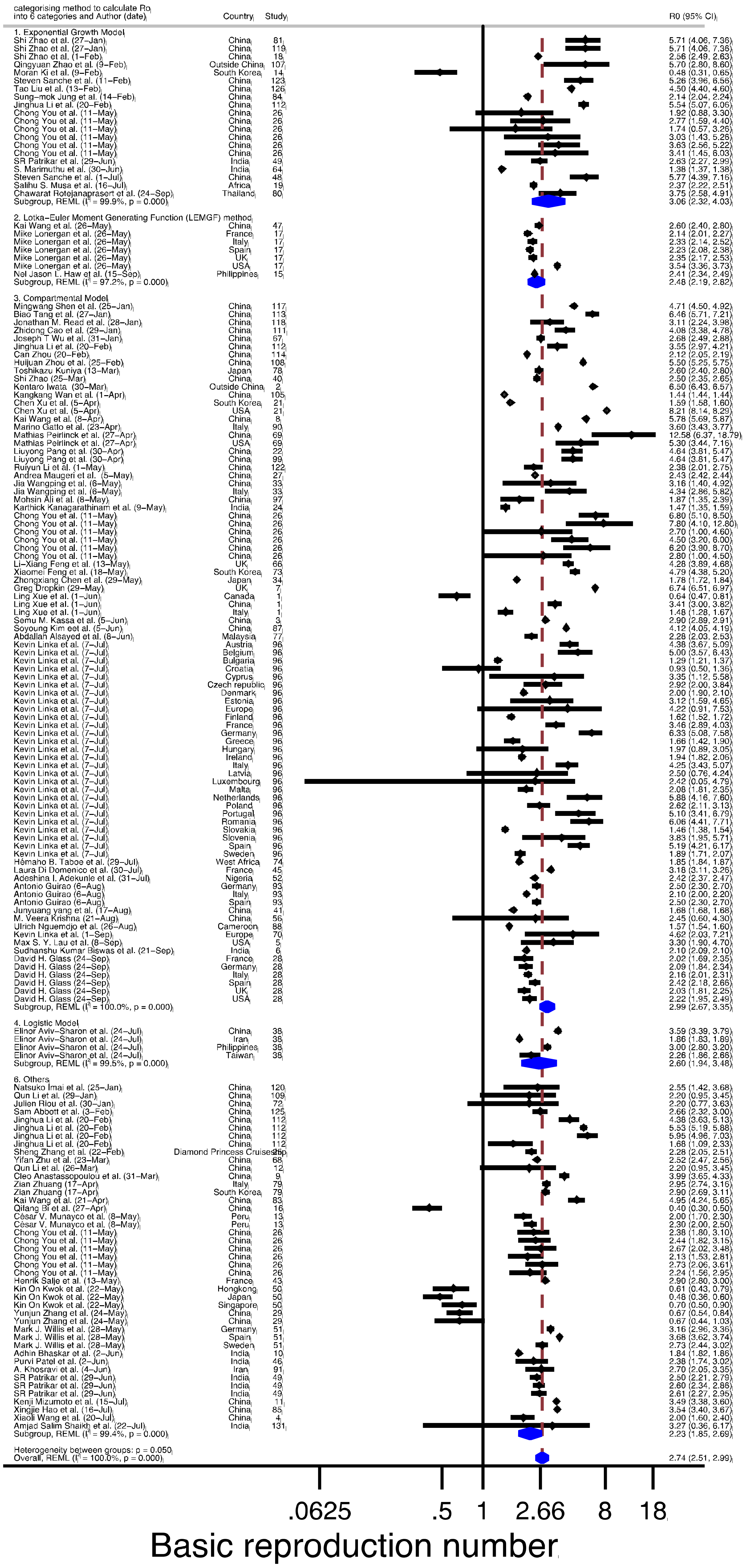

Figure S2. Sub-group analysis of basic reproduction number by duration of data

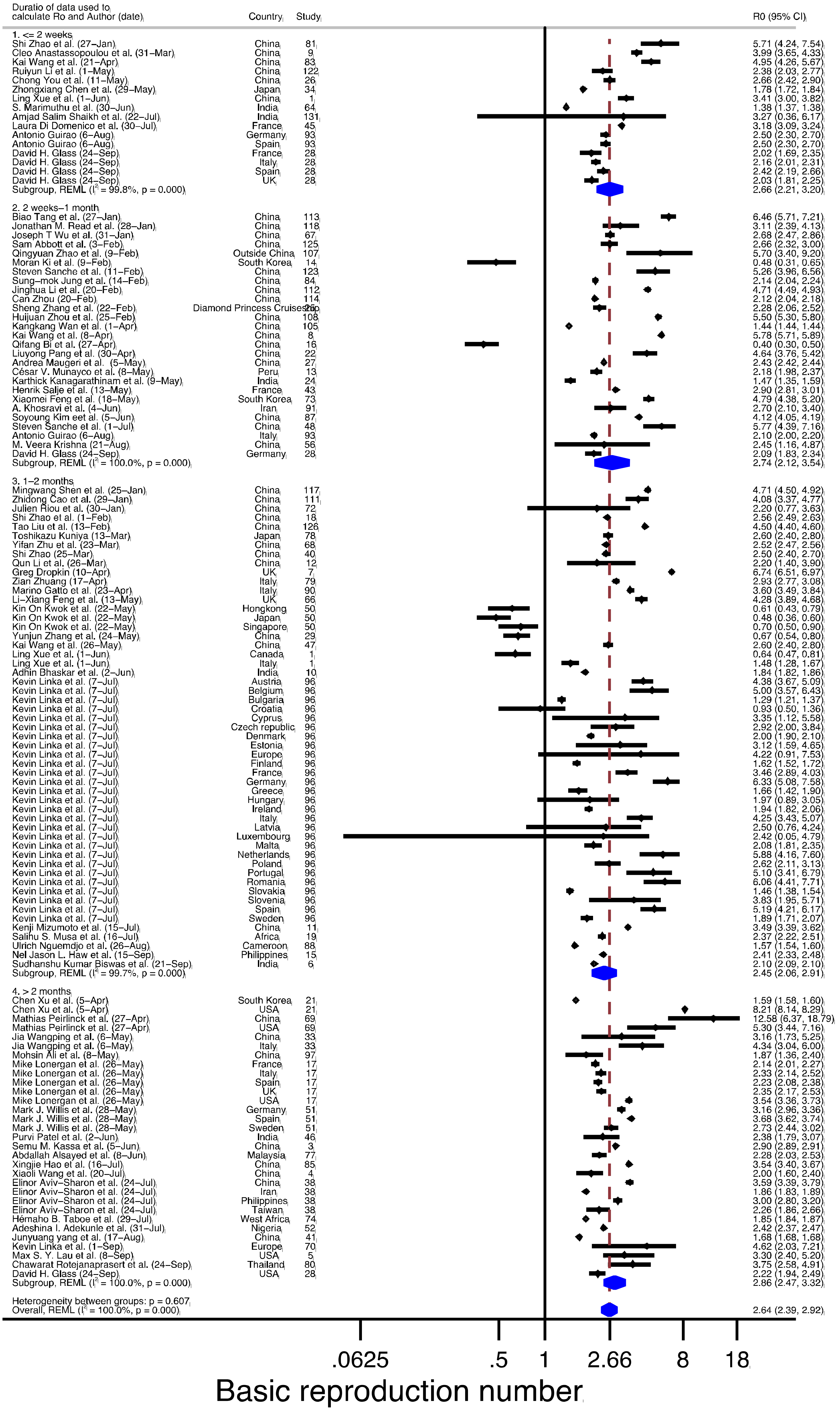

Figure S3. Sub-group analysis of basic reproduction number by last month of data

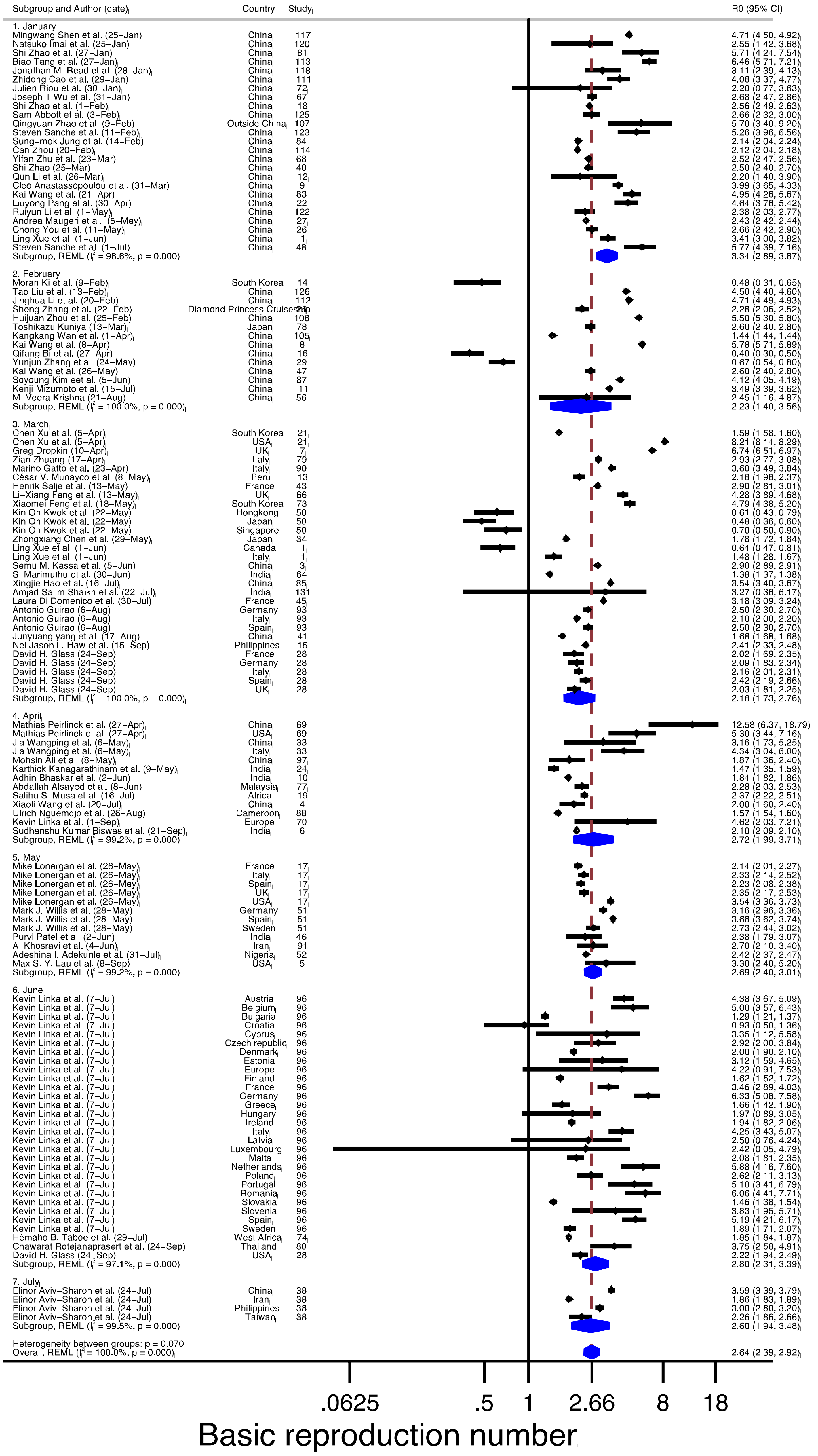

Figure S4. Sub-group analysis of basic reproduction number by month of publication

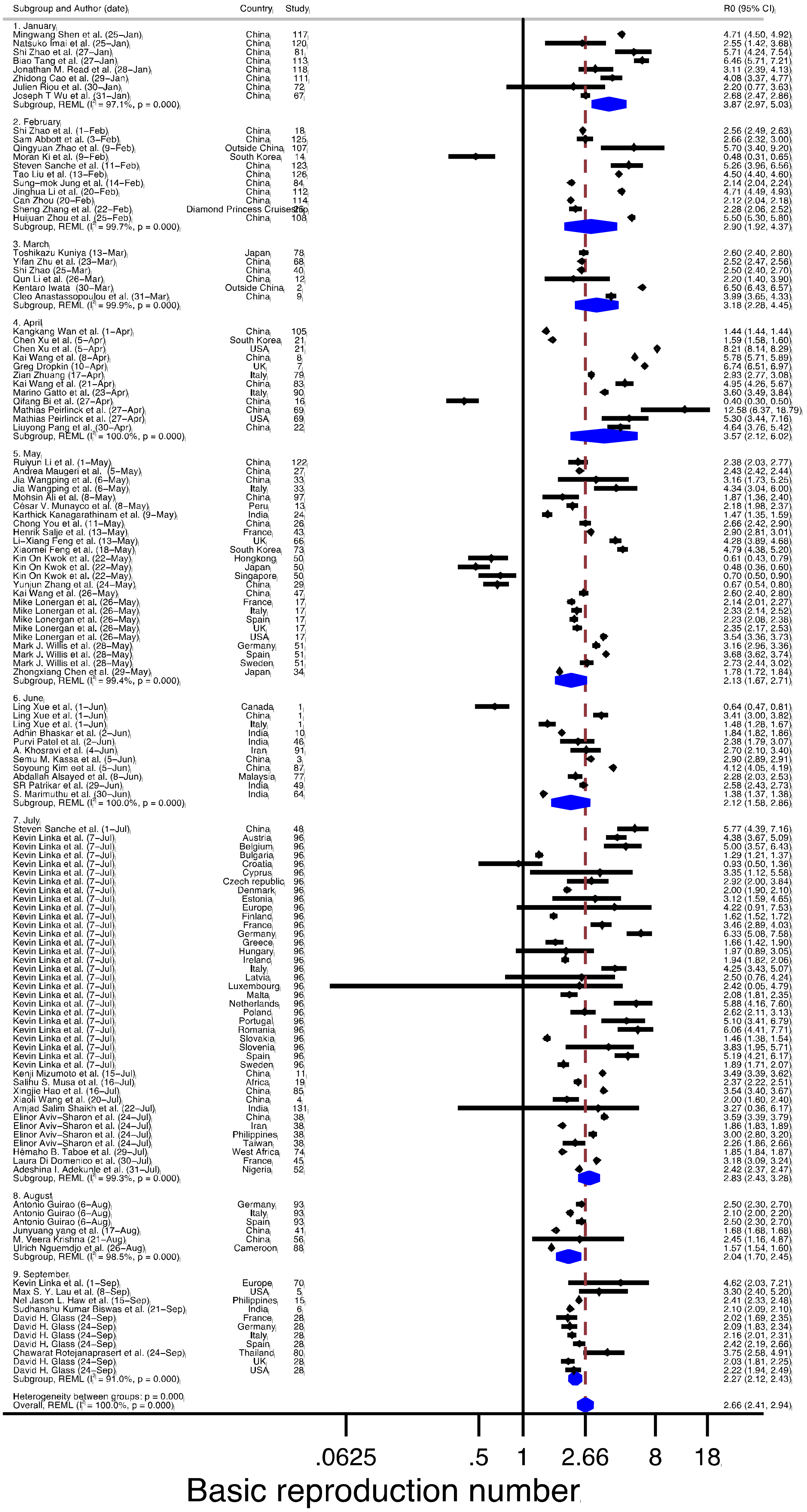

Figure S5. Sub-group analysis of basic reproduction number by country

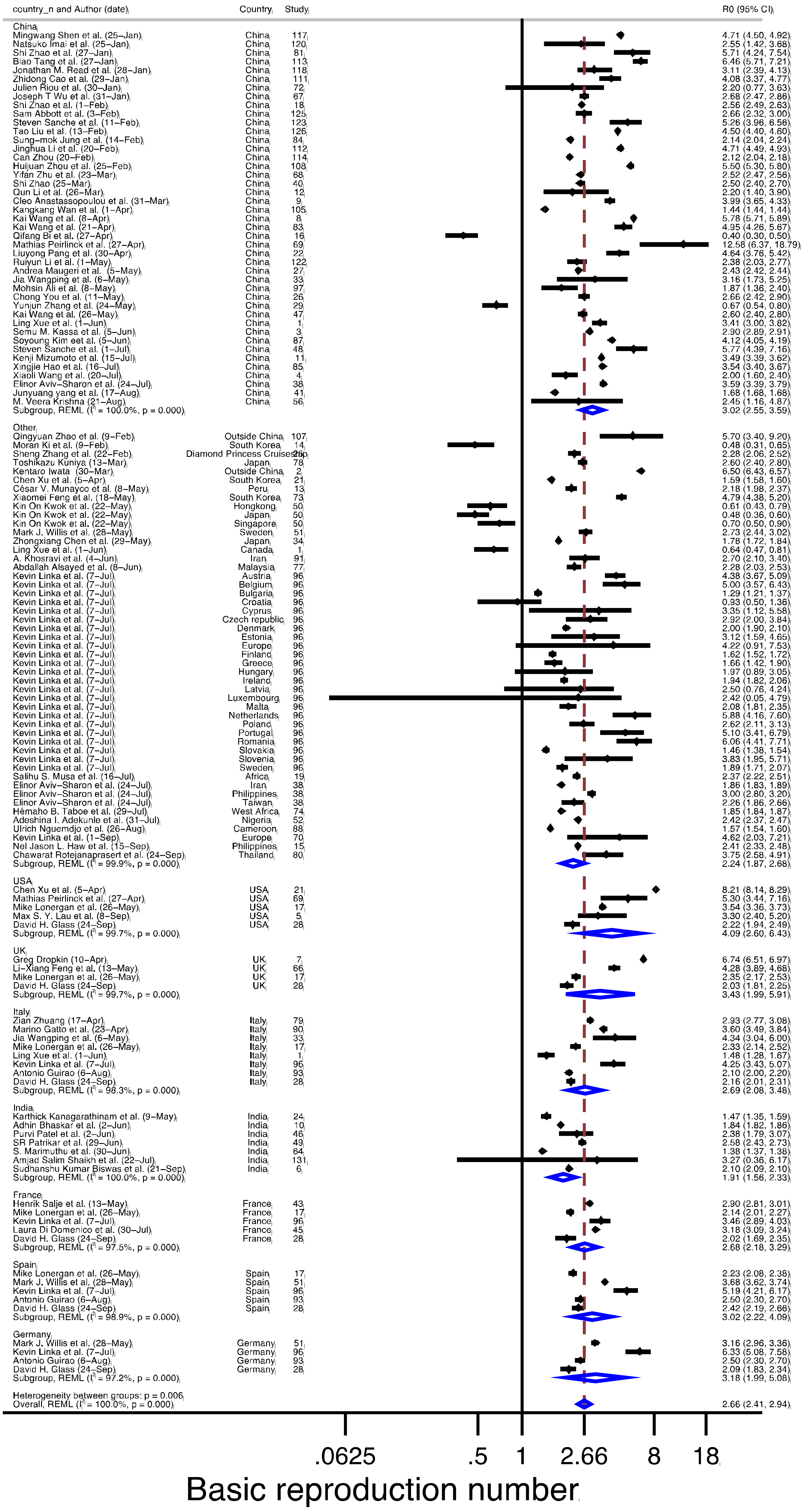

Figure S6. Sub-group analysis of basic reproduction number by type of central estimate

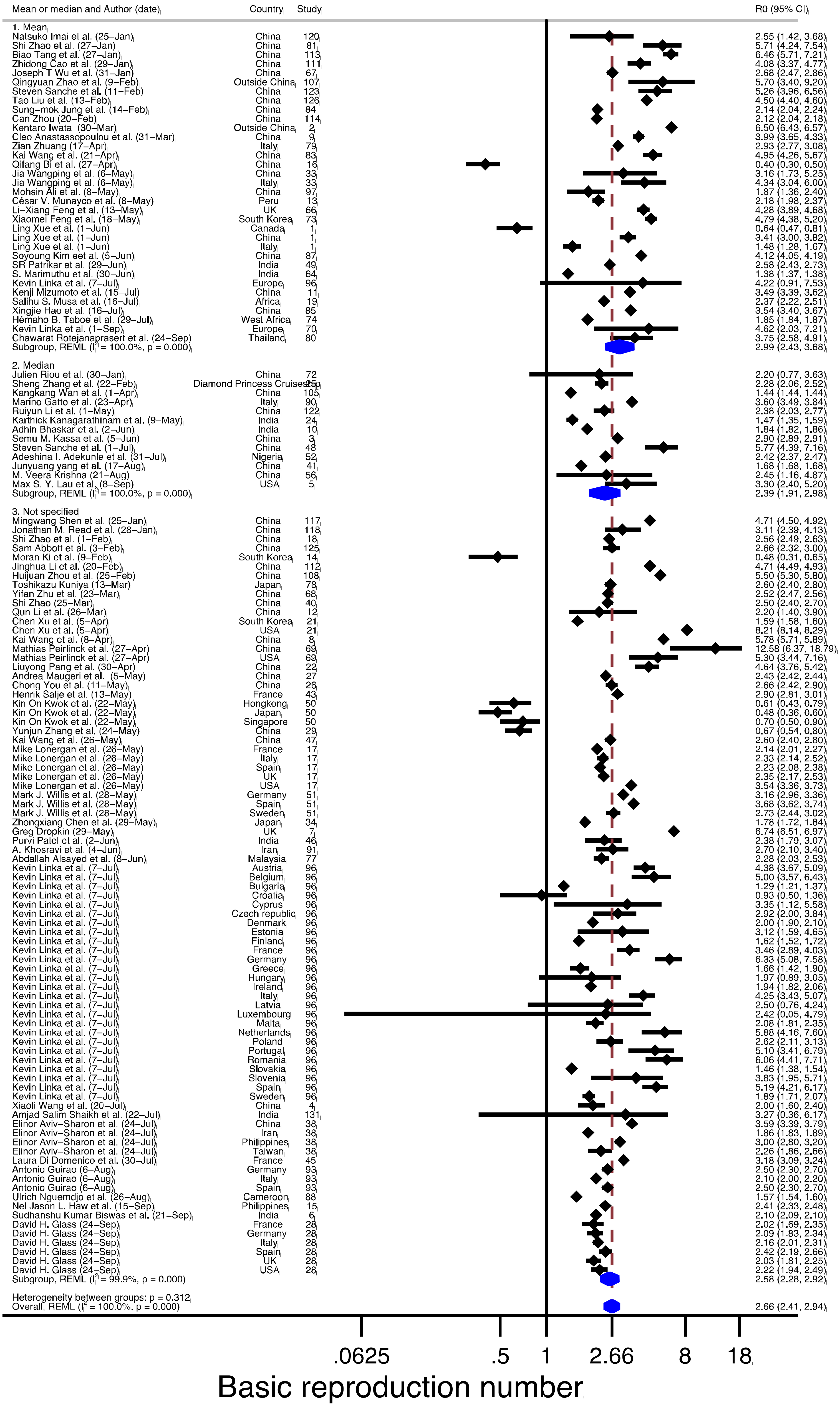

Figure S7. Sub-group analysis of basic reproduction number by location in China

Whether the location is Hubei, Wuhan, Outside Hubei or overall and Author (date)	Country	city	Study	R0 (95% CI)
1. Wuhan				
Ling Xue et al. (1–Jun)	China	Wuhan	1	3.41 (3.00, 3.82)
Kenji Mizumoto et al. (15–Jul)	China	Wuhan	11	3.49 (3.39, 3.62)
Qun Li et al. (26–Mar)	China	Wuhan	12	2.20 (1.40, 3.90)
Liuyong Pang et al. (30–Apr)	China	Wuhan	22	4.64 (3.76, 5.42)
Shi Zhao (25–Mar)	China	Wuhan	40	2.50 (2.40, 2.70)
Steven Sanche et al. (1–Jul)	China	Wuhan	48	5.77 (4.39, 7.16)
Mohsin Ali et al. (8–May)	China	Wuhan	97	1.87 (1.36, 2.40)
Jinghua Li et al. (20–Feb)	China	Wuhan	112	4.71 (4.49, 4.93)
Subgroup, REML (I^2 = 97.8%, p = 0.000)				3.40 (2.61, 4.44)
2. Hubei				
Semu M. Kassa et al. (5–Jun)	China	Hubei	3	2.90 (2.89, 2.91)
Cleo Anastassopoulou et al. (31–Mar)	China	Hubei	9	3.99 (3.65, 4.33)
Subgroup, REML (I^2 = 98.2%, p = 0.000)				3.39 (2.48, 4.64)
3. Outside Hubei				
Xiaoli Wang et al. (20–Jul)	China	Beijing	4	2.00 (1.60, 2.40)
Qifang Bi et al. (27–Apr)	China	Shenzhen	16	0.40 (0.30, 0.50)
Chong You et al. (11–May)	China	Shanghai	26	2.66 (2.42, 2.90)
Yunjun Zhang et al. (24–May)	China	Tianjin	29	0.67 (0.54, 0.80)
Jia Wangping et al. (6–May)	China	Hunan	33	3.16 (1.73, 5.25)
Kai Wang et al. (26–May)	China	Shenzhen	47	2.60 (2.40, 2.80)
Subgroup, REML (I^2 = 98.5%, p = 0.000)				1.50 (0.76, 2.96)
4. Overall				
Kai Wang et al. (8–Apr)	China	Overall	8	5.78 (5.71, 5.89)
Shi Zhao et al. (1–Feb)	China	Mainland	18	2.56 (2.49, 2.63)
Andrea Maugeri et al. (5–May)	China	Overall	27	2.43 (2.42, 2.44)
Elinor Aviv–Sharon et al. (24–Jul)	China	Mainland	38	3.59 (3.39, 3.79)
Junyuang yang et al. (17–Aug)	China	Mainland	41	1.68 (1.68, 1.68)
M. Veera Krishna (21–Aug)	China		56	2.45 (1.16, 4.87)
Joseph T Wu et al. (31–Jan)	China		67	2.68 (2.47, 2.86)
Yifan Zhu et al. (23–Mar)	China		68	2.52 (2.47, 2.56)
Mathias Peirlinck et al. (27–Apr)	China		69	12.58 (6.37, 18.79)
Julien Riou et al. (30–Jan)	China		72	2.20 (0.77, 3.63)
Shi Zhao et al. (27–Jan)	China		81	5.71 (4.24, 7.54)
Kai Wang et al. (21–Apr)	China		83	4.95 (4.26, 5.67)
Sung–mok Jung et al. (14–Feb)	China		84	2.14 (2.04, 2.24)
Xingjie Hao et al. (16–Jul)	China		85	3.54 (3.40, 3.67)
Soyoung Kim eet al. (5–Jun)	China		87	4.12 (4.05, 4.19)
Kangkang Wan et al. (1–Apr)	China		105	1.44 (1.44, 1.44)
Huijuan Zhou et al. (25–Feb)	China		108	5.50 (5.30, 5.80)
Zhidong Cao et al. (29–Jan)	China		111	4.08 (3.37, 4.77)
Biao Tang et al. (27–Jan)	China		113	6.46 (5.71, 7.21)
Can Zhou (20–Feb)	China		114	2.12 (2.04, 2.18)
Mingwang Shen et al. (25–Jan)	China		117	4.71 (4.50, 4.92)
Jonathan M. Read et al. (28–Jan)	China		118	3.11 (2.39, 4.13)
Natsuko Imai et al. (25–Jan)	China		120	2.55 (1.42, 3.68)
Ruiyun Li et al. (1–May)	China		122	2.38 (2.03, 2.77)
Steven Sanche et al. (11–Feb)	China		123	5.26 (3.96, 6.56)
Sam Abbott et al. (3–Feb)	China		125	2.66 (2.32, 3.00)
Tao Liu et al. (13–Feb)	China		126	4.50 (4.40, 4.60)
Subgroup, REML (I^2 = 100.0%, p = 0.000)				3.39 (2.84, 4.04)
Heterogeneity between groups: p = 0.150				
Overall, REML (I^2 = 100.0%, p = 0.000)				3.02 (2.55, 3.59)

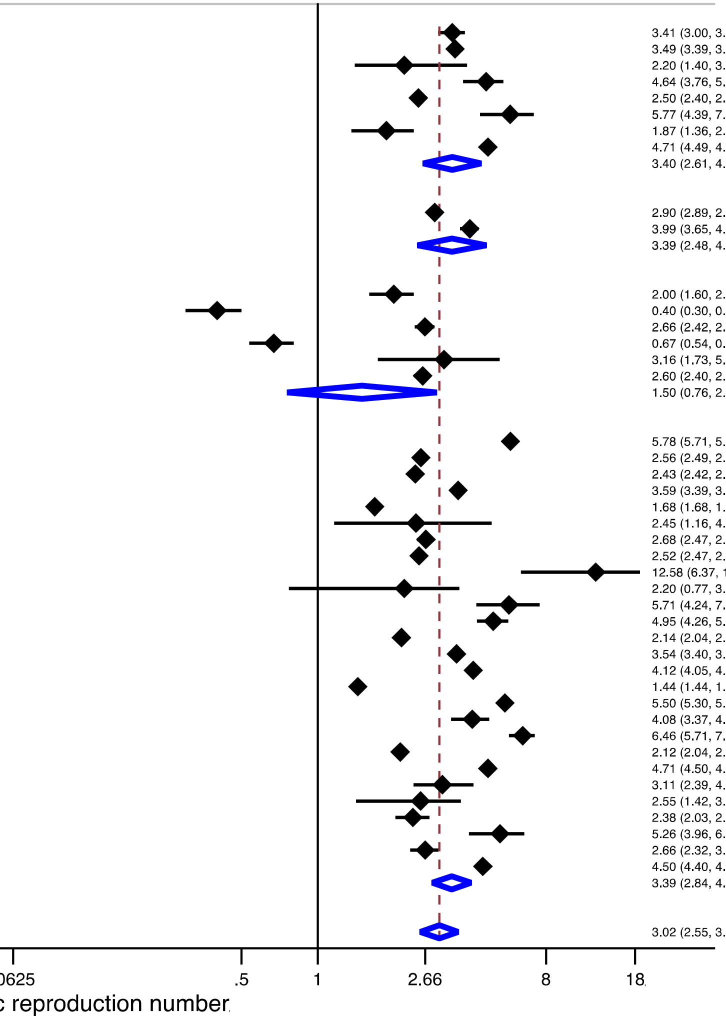

NOTE: Weights and between–subgroup heterogeneity test are from random–effects model

14

Figure S8. Sub-group analysis of basic reproduction number by continent

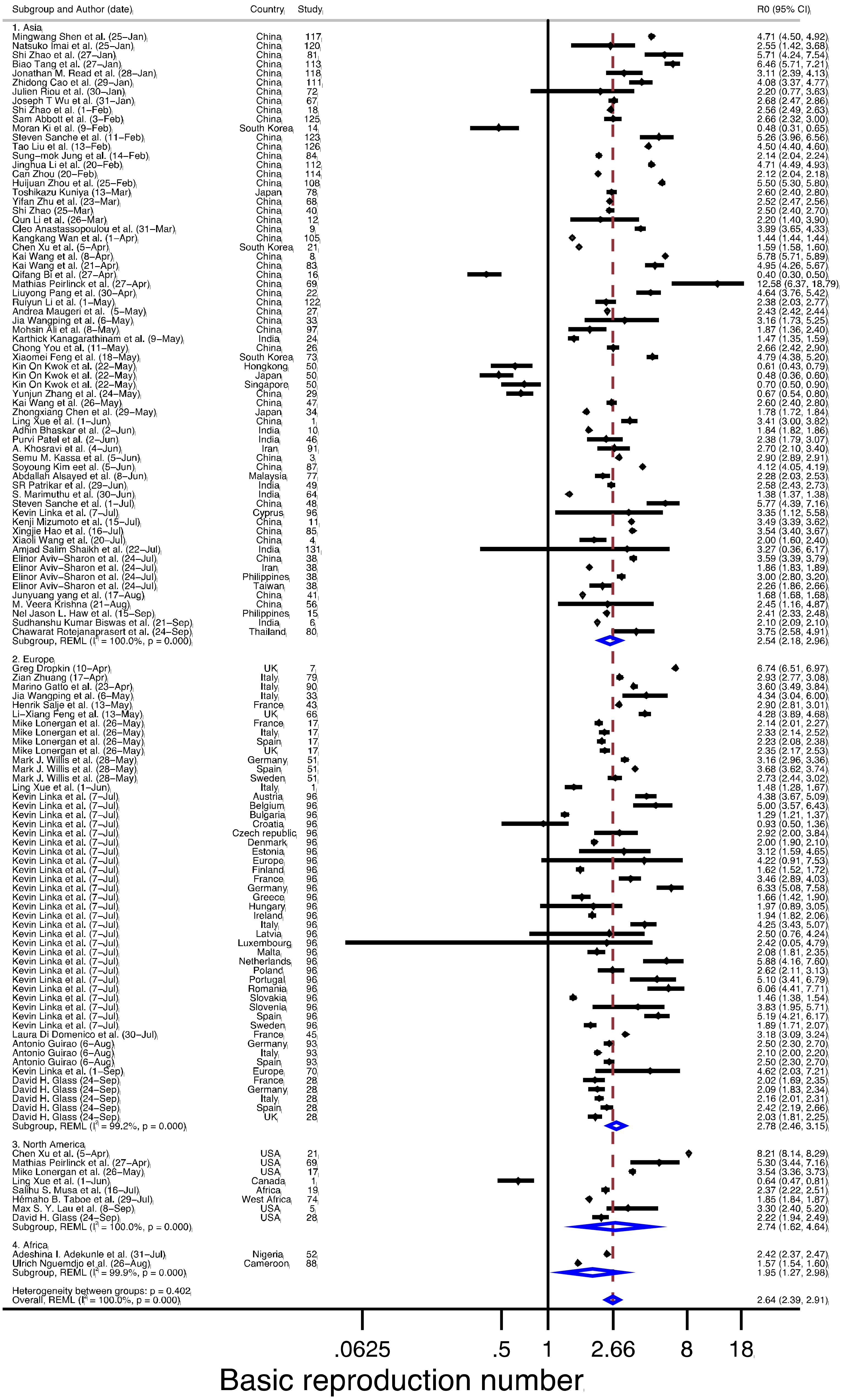

Figure S9. Sensitivity analysis of basic reproduction number excluding studies with $R_0 \leq 1$

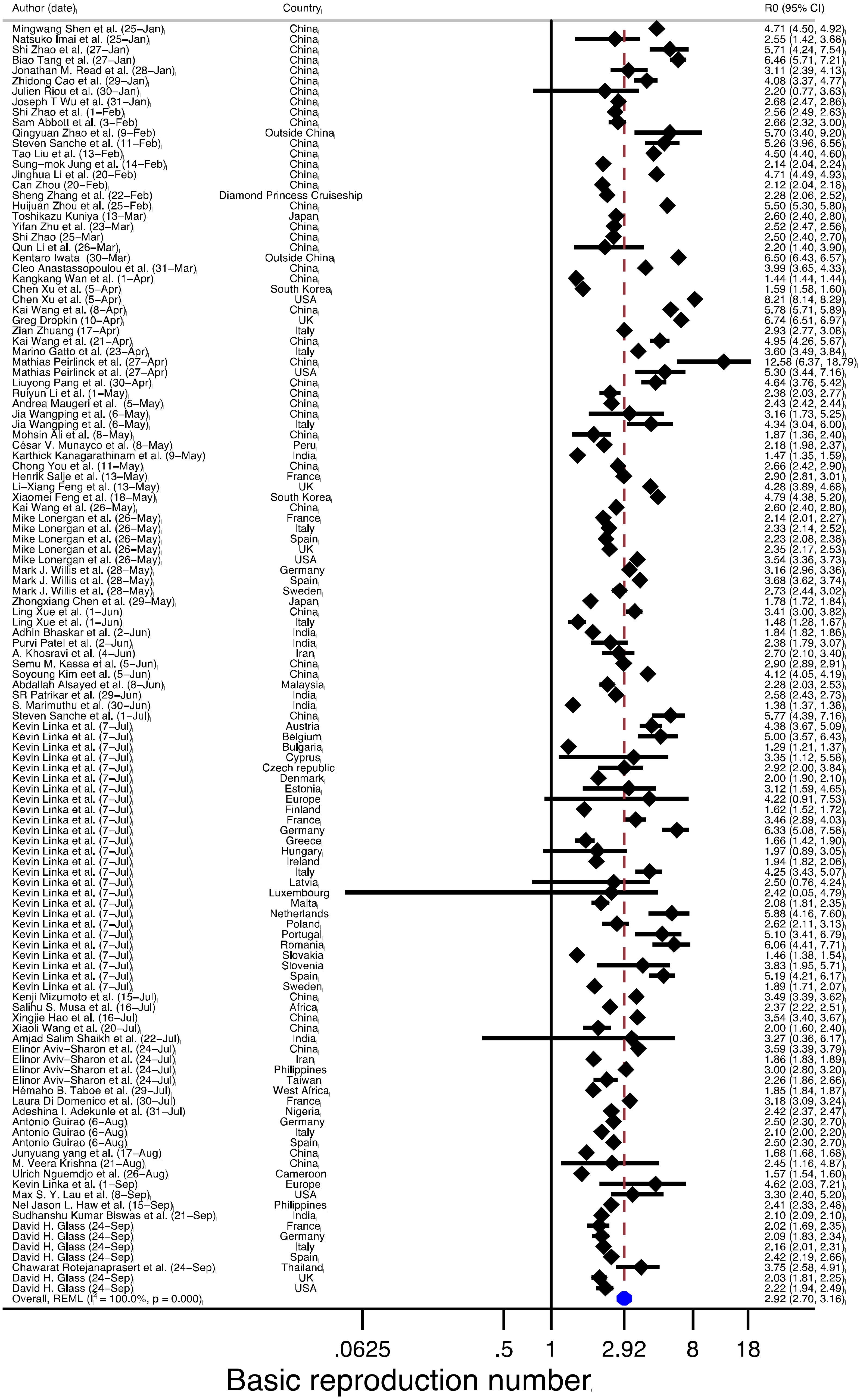

Figure S10. Sensitivity analysis of basic reproduction number excluding fair or low-quality studies

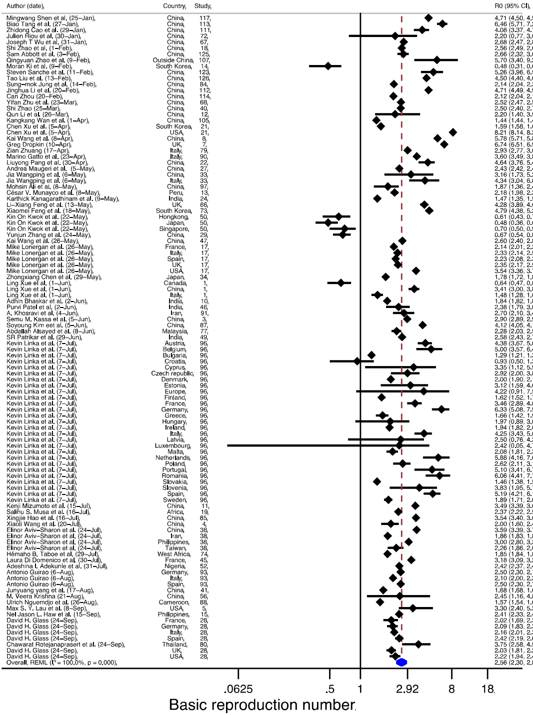

17

Supplementary tables

Table S1. Descriptive characteristics including quality assessment of studies with basic reproduction number included in meta-analysis

(Please refer to the excel file)

Table S2. Result of influence analysis for basic reproduction number

Study no. omitted	Author	Estimate	95% Confidence interval
1	Ling Xue et al.	2.68	(2.42–2.95)
1	Ling Xue et al.	2.66	(2.41–2.94)
1	Ling Xue et al.	2.69	(2.44–2.97)
2	Kentaro Iwata	2.64	(2.40–2.92)
3	Semu M. Kassa et al.	2.66	(2.41–2.94)
4	Xiaoli Wang et al.	2.67	(2.42–2.95)
5	Max S. Y. Lau et al.	2.66	(2.41–2.94)
6	Sudhanshu Kumar Biswas et al.	2.67	(2.42–2.95)
7	Greg Dropkin	2.64	(2.40–2.92)
8	Kai Wang et al.	2.65	(2.40–2.92)
9	Cleo Anastassopoulou et al.	2.65	(2.40–2.93)
10	Adhin Bhaskar et al.	2.67	(2.42–2.95)
11	Kenji Mizumoto et al.	2.66	(2.41–2.93)
12	Qun Li et al.	2.67	(2.41–2.94)
13	César V. Munayco et al.	2.67	(2.41–2.95)
14	Moran Ki et al.	2.70	(2.45–2.97)
15	Nel Jason L. Haw et al.	2.67	(2.41–2.94)
16	Qifang Bi et al.	2.70	(2.46–2.97)
17	Mike Lonergan et al.	2.67	(2.41–2.94)
17	Mike Lonergan et al.	2.67	(2.41–2.94)
17	Mike Lonergan et al.	2.67	(2.41–2.95)
17	Mike Lonergan et al.	2.66	(2.41–2.93)
17	Mike Lonergan et al.	2.67	(2.42–2.95)
18	Shi Zhao et al.	2.66	(2.41–2.94)
19	Salihu S. Musa et al.	2.67	(2.41–2.94)
21	Chen Xu et al.	2.64	(2.39–2.91)
21	Chen Xu et al.	2.67	(2.42–2.95)
22	Liuyong Pang et al.	2.65	(2.40–2.93)
24	Karthick Kanagarathinam et al.	2.68	(2.42–2.95)
25	Sheng Zhang et al.	2.67	(2.41–2.95)
26	Chong You et al.	2.66	(2.41–2.94)
27	Andrea Maugeri et al.	2.66	(2.41–2.94)
28	David H. Glass	2.67	(2.42–2.95)
28	David H. Glass	2.67	(2.41–2.95)
28	David H. Glass	2.67	(2.42–2.95)
28	David H. Glass	2.67	(2.41–2.95)
28	David H. Glass	2.67	(2.42–2.95)
28	David H. Glass	2.67	(2.41–2.94)
29	Yunjun Zhang et al.	2.69	(2.44–2.97)
33	Jia Wangping et al.	2.66	(2.41–2.94)
33	Jia Wangping et al.	2.65	(2.40–2.93)
34	Zhongxiang Chen et al.	2.67	(2.42–2.95)
38	Elinor Aviv-Sharon et al.	2.66	(2.41–2.93)
38	Elinor Aviv-Sharon et al.	2.66	(2.41–2.94)
38	Elinor Aviv-Sharon et al.	2.67	(2.42–2.95)
38	Elinor Aviv-Sharon et al.	2.67	(2.41–2.95)
40	Shi Zhao	2.66	(2.41–2.94)
41	Junyuang yang et al.	2.67	(2.42–2.95)
43	Henrik Salje et al.	2.66	(2.41–2.94)
45	Laura Di Domenico et al.	2.66	(2.41–2.94)
46	Purvi Patel et al.	2.67	(2.41–2.94)
47	Kai Wang et al.	2.66	(2.41–2.94)
48	Steven Sanche et al.	2.65	(2.40–2.92)
49	SR Patrikar et al.	2.66	(2.41–2.94)
50	Kin On Kwok et al.	2.69	(2.44–2.97)
50	Kin On Kwok et al.	2.69	(2.44–2.97)
50	Kin On Kwok et al.	2.70	(2.45–2.97)

51	Mark J. Willis et al.	2.66	(2.41–2.94)
51	Mark J. Willis et al.	2.66	(2.41–2.94)
51	Mark J. Willis et al.	2.66	(2.40–2.93)
52	Adeshina I. Adekunle et al.	2.67	(2.41–2.94)
56	M. Veera Krishna	2.66	(2.41–2.94)
64	S. Marimuthu et al.	2.68	(2.43–2.96)
66	Li-Xiang Feng et al.	2.65	(2.40–2.93)
67	Joseph T Wu et al.	2.66	(2.41–2.94)
68	Yifan Zhu et al.	2.66	(2.41–2.94)
69	Mathias Peirlinck et al.	2.64	(2.39–2.90)
69	Mathias Peirlinck et al.	2.65	(2.40–2.92)
70	Kevin Linka et al.	2.65	(2.40–2.93)
72	Julien Riou et al.	2.67	(2.41–2.94)
73	Xiaomei Feng et al.	2.65	(2.40–2.93)
74	Hémaho B. Taboe et al.	2.67	(2.42–2.95)
77	Abdallah Alsayed et al.	2.67	(2.41–2.95)
78	Toshikazu Kuniya	2.66	(2.41–2.94)
79	Zian Zhuang	2.66	(2.41–2.94)
80	Chawarat Rotejanaprasert et al.	2.66	(2.40–2.93)
81	Shi Zhao et al.	2.65	(2.40–2.92)
83	Kai Wang et al.	2.65	(2.40–2.93)
84	Sung-mok Jung et al.	2.67	(2.42–2.95)
85	Xingjie Hao et al.	2.66	(2.41–2.93)
87	Soyoung Kim eet al.	2.65	(2.40–2.93)
88	Ulrich Nguemdjo et al.	2.67	(2.42–2.95)
90	Marino Gatto et al.	2.66	(2.41–2.93)
91	A. Khosravi et al.	2.66	(2.41–2.94)
93	Antonio Guirao	2.66	(2.41–2.94)
93	Antonio Guirao	2.67	(2.42–2.95)
93	Antonio Guirao	2.66	(2.41–2.94)
96	Kevin Linka et al.	2.66	(2.41–2.94)
96	Kevin Linka et al.	2.64	(2.40–2.92)
96	Kevin Linka et al.	2.67	(2.42–2.95)
96	Kevin Linka et al.	2.67	(2.42–2.95)
96	Kevin Linka et al.	2.66	(2.41–2.93)
96	Kevin Linka et al.	2.66	(2.41–2.94)
96	Kevin Linka et al.	2.65	(2.40–2.92)
96	Kevin Linka et al.	2.67	(2.42–2.95)
96	Kevin Linka et al.	2.67	(2.42–2.95)
96	Kevin Linka et al.	2.66	(2.41–2.94)
96	Kevin Linka et al.	2.66	(2.41–2.94)
96	Kevin Linka et al.	2.66	(2.41–2.94)
96	Kevin Linka et al.	2.68	(2.43–2.96)
96	Kevin Linka et al.	2.65	(2.40–2.92)
96	Kevin Linka et al.	2.65	(2.40–2.92)
96	Kevin Linka et al.	2.66	(2.41–2.94)
96	Kevin Linka et al.	2.67	(2.42–2.95)
96	Kevin Linka et al.	2.65	(2.40–2.93)
96	Kevin Linka et al.	2.65	(2.40–2.93)
96	Kevin Linka et al.	2.68	(2.43–2.96)
96	Kevin Linka et al.	2.65	(2.40–2.93)
96	Kevin Linka et al.	2.65	(2.40–2.93)
96	Kevin Linka et al.	2.66	(2.41–2.93)
96	Kevin Linka et al.	2.67	(2.42–2.95)
96	Kevin Linka et al.	2.66	(2.41–2.94)
96	Kevin Linka et al.	2.67	(2.42–2.95)
96	Kevin Linka et al.	2.68	(2.42–2.95)
97	Mohsin Ali et al.	2.67	(2.42–2.95)
105	Kangkang Wan et al.	2.68	(2.42–2.95)
107	Qingyuan Zhao et al.	2.65	(2.40–2.92)

108	Huijuan Zhou et al.	2.65	(2.40–2.92)
111	Zhidong Cao et al.	2.65	(2.40–2.93)
112	Jinghua Li et al.	2.65	(2.40–2.93)
113	Biao Tang et al.	2.64	(2.40–2.92)
114	Can Zhou	2.67	(2.42–2.95)
117	Mingwang Shen et al.	2.65	(2.40–2.93)
118	Jonathan M. Read et al.	2.66	(2.41–2.94)
120	Natsuko Imai et al.	2.66	(2.41–2.94)
122	Ruiyun Li et al.	2.67	(2.41–2.94)
123	Steven Sanche et al.	2.65	(2.40–2.92)
125	Sam Abbott et al.	2.66	(2.41–2.94)
126	Tao Liu et al.	2.65	(2.40–2.93)
131	Amjad Salim Shaikh et al.	2.66	(2.41–2.94)
Combined		2.66	2.41

Table S3. Narrative synthesis including quality assessment of the studies with basic reproduction number included in the review

(Please refer to the excel file
