# Supplementary material for: Reliability of Early Estimates of the Basic Reproduction Number of COVID-19: A Systematic Review and Meta-Analysis
Source: Int J Environ Res Public Health. 2022 Sep 15;19(18):11613. doi: 10.3390/ijerph191811613 (PMC9517346; doi:10.3390/ijerph191811613)
Supplement: Supplementary file 1 [file ijerph-19-11613-s001.zip › Supplementary file S2.pdf]

Citation

Bibha Dhungel, Md. Shafiur Rahman, Md. Mahfuzur Rahman, Aliza KC Bhandari, Le Mai Phuong, Nushrat Alam Biva, Stuart Gilmour. A systematic review and meta-analysis of the transmissibility and severity of COVID-19. PROSPERO 2021 CRD42021279514 Available from:
https://www.crd.york.ac.uk/prospero/display_record.php?ID=CRD42021279514

Review question

1. What is the basic and effective reproduction number (Ro) of COVID-19 in different countries?
2. What is the impact of study-related factors (methods, assumptions, study location, amount of data, study period) on the estimated Ro?
3. What is the case fatality rate of COVID-19 across the countries?
4. What is the prevalence of COVID-19?
5. What is the mortality rate of COVID-19?
- What is the number of deaths and mortality rate directly attributable to COVID-19?
- What is the number of excess deaths and excess mortality rate, directly and indirectly, attributable to COVID-19?
- How is the projection of mortality/mortality rate of COVID-19 in early studies different from the actual outcome?
6. What is the impact of the lockdowns?
- What is the incidence rate and attack rate before and 7 days after lockdown?
- By what percent did the number of cases got reduced in 7 days after lockdown compared to the baseline scenario without lockdown?
- What is the serial interval between successive cases?

Searches

All COVID-19 case-related studies with titles and abstracts in any language published between 1st December 2019 and 31st September 2020 will be searches.
A final search will be made again to include newly published studies.

The search will be performed in several electronic databases: LitCovid, PubMed, MEDLINE, CINAHL, PsycINFO, EMBASE, COVID-19 database by the World Health Organization, British Nursing Index, Coronavirus Research Database, Web of Science, and OVID Global Health.
Non-English electronic databases such as CiNii for the Japanese language will also be searched.
In addition, preprint databases including arXiv, bioRxiv, and medRxiv will be searched to identify additional studies.
Moreover, Google Scholar and the reference list of the relevant articles will be searched to find additional relevant studies.

Types of study to be included

We will include primary studies reporting on either COVID-19-related mortality, the incidence of disease, infection rates or reproduction rates.

Inclusion criteria:
- Observational studies on human beings published in peer-reviewed journals or on pre-print servers;
- Reports by government agencies providing official numbers of cases and deaths;
- Simulation studies.

Exclusion criteria:
- We will exclude opinion pieces (such as editorials and letters to the editor), non-original reports (such as systematic reviews and meta-analysis), and original reports of lab-based experimental studies (including in vitro and in vivo studies, animal studies);

- We will exclude studies focusing on other coronaviruses and other diseases.

Condition or domain being studied

Wuhan detected an outbreak of a novel coronavirus disease 2019 (COVID-19) in December 2019. The symptoms of COVID-19 range from mild fever, cough, and shortness of breath and may later lead to pneumonia and respiratory failure.

Participants/population

The study population includes people of any age or gender residing in any geographic area with a confirmed diagnosis of COVID-19.

Intervention(s), exposure(s)

We will analyse the impact of lockdown or travel restrictions on incidence rate, the number of cases, serial interval, and reproduction number of COVID-19.

Comparator(s)/control

Incidence rate, number of cases, serial interval and reproduction number of COVID-19 before the lockdown or travel restrictions.

Main outcome(s)

The basic or effective reproduction number of COVID-19 over time and geographical space.

Measures of effect

Basic reproduction number.
Effective reproduction number.

Additional outcome(s)

1. Case-fatality rate (CFR) of COVID-19.
2. The COVID-19 infection rate.
3. The incidence of COVID-19 cases.
4. Mortality directly attributable to COVID-19.
5. Excess mortality directly or indirectly attributable to COVID-19.
6. The impact of lockdown on COVID-19.

Measures of effect

1. The case fatality rate of confirmed COVID-19 cases.
2. The COVID-19 infection rate in the general population.
3. The incidence of confirmed COVID-19 cases in the general population.
4. The absolute number of deaths with a confirmed diagnosis of COVID-19
- COVID-19 mortality rate (confirmed COVID-19 cases).
5. Difference between the absolute number of deaths in a general population at any point during the period of the COVID-19 outbreak (starting in January 2020) and the average number of deaths in the general population in the same period in previous years
- Difference between mortality rate in a general population at any point during the period of the COVID-19 outbreak (starting in January 2020) and the mortality rate in the general population in the same period in previous years.
6. The difference between early projection of disease incidence and the incidence rate after lockdown; the attack rate; serial interval; changes/reduction in incidence, changes/reduction in mortality rate.

Data extraction (selection and coding)

Study selection:
Titles and abstracts screening will be done independently by three investigators using Rayyan QCRI. Full-text papers of the studies selected in the title/abstract screening will be screened by two independent investigators. Any disagreements on the included articles will be resolved via consensus.

Data extraction:

Two investigators will independently perform the data extraction on the studies selected for inclusion during full-text screening. Any disagreements will be resolved by consensus or after discussing with the principal investigator. A standardized data extraction form will be prepared to capture the following information: study identifiers (Journal name, DOI), title, author, author affiliation, month and year of publication, study period, types of data used, study design, study method, location, sample size, model used, susceptible population, risk of bias, the characteristics of the participants, COVID-19 deaths and mortality rate, basic or effective reproduction number with confidence interval and/or standard deviation (or other relevant statistics), case fatality rate, excess deaths, and excess mortality rate, directly and indirectly, attributable to COVID-19, incidence/prevalence rate of COVID-19, infection rate/attack rate and study quality assessment.

Risk of bias (quality) assessment

We will use the risk of bias tool to perform critical appraisal of included studies, as recommended by the Cochrane Collaboration (Higgins JPT et al., 2020). We will use the National Institutes of Health (NIH) quality assessment tool to assess study quality. Additionally, the quality of each included non-randomised study will be assessed using the Newcastle-Ottawa scale in terms of selection (0-4 stars), comparability (0-2 stars) of the groups, and ascertainment of outcome (0-3 stars) with a maximum of 9 stars representing highest methodological quality (George Wells et al., 2000).

Strategy for data synthesis

We will conduct a narrative review for studies without confidence intervals or standard errors. When two or more studies are available for a study outcome, we will perform a meta-analysis.
We will use a fixed-effect model to get a summary effect if the outcome is measured using different models. We will then estimate summary effect sizes using a random-effects meta-analysis model and will show the extent of heterogeneity (I^2 statistic, $?^2$, and Cochran's Q test) across studies.
Leave-one-out meta-analysis will be used to determine the impact of studies on the pooled estimate. The sensitivity analysis will be conducted excluding the most influential studies.
The Begg's funnel plot and the asymmetry test (Egger's test) will be used to assess publication bias. The trim-and-fill approach will be used to address publication bias, if any.

Analysis of subgroups or subsets

Subgroup analysis will be performed according to study design/method, model, time duration, country, geographic region, and country-income category. It is necessary as we expect the effect sizes to vary substantially depending on the various intervention strategies in different geographic regions over time. We expect heterogeneity depending on the study method or mathematical model used as well. We will conduct meta-regression to identify the effects of study-level covariates.

Contact details for further information

Bibha Dhungel
18mp201@slcn.ac.jp

Organisational affiliation of the review

St. Luke's International University

Review team members and their organisational affiliations

Ms Bibha Dhungel. St. Luke's International University
Ms Md. Shafiur Rahman. Hamamatsu University School of Medicine
Mr Md. Mahfuzur Rahman. Global Public Health Research Foundation
Ms Aliza KC Bhandari. St. Luke's International University
Ms Le Mai Phuong. St. Luke's International University
Ms Nushrat Alam Biva. St. Luke's International University
Professor Stuart Gilmour. St. Luke's International University

Type and method of review

Epidemiologic, Meta-analysis, Systematic review

Anticipated or actual start date

15 October 2020

Anticipated completion date

14 February 2022

Funding sources/sponsors
None

Conflicts of interest

Language
English

Country
Bangladesh, Japan

Stage of review
Review Ongoing

Subject index terms status
Subject indexing assigned by CRD

Subject index terms
Basic Reproduction Number; Communicable Disease Control; Communicable Diseases; COVID-19; Disease Progression; Disease Transmission, Infectious; Humans; Incidence; Mortality; Prevalence; Public Health; SARS-CoV-2

Date of registration in PROSPERO
27 September 2021

Date of first submission
26 September 2021

Stage of review at time of this submission

**Stage**	**Started**	**Completed**
Preliminary searches	Yes	Yes
Piloting of the study selection process	Yes	Yes
Formal screening of search results against eligibility criteria	Yes	Yes
Data extraction	No	No
Risk of bias (quality) assessment	No	No
Data analysis	No	No

The record owner confirms that the information they have supplied for this submission is accurate and complete and they understand that deliberate provision of inaccurate information or omission of data may be construed as scientific misconduct.

The record owner confirms that they will update the status of the review when it is completed and will add publication details in due course.

Versions
27 September 2021
