# Supplementary material for: Reliability of Early Estimates of the Basic Reproduction Number of COVID-19: A Systematic Review and Meta-Analysis
Source: Int J Environ Res Public Health. 2022 Sep 15;19(18):11613. doi: 10.3390/ijerph191811613 (PMC9517346; doi:10.3390/ijerph191811613)
Supplement: Supplementary file 1 [file ijerph-19-11613-s001.zip › Supplementary file S4.pdf]

Data extraction form

Study ID *(study number eg. 1, 2..)*	

General information

Date form completed *(dd/mm/yyyy)*	
Name of person extracting data	
Notes:	

Study details

Study title	
DOI	
Journal	
Author	
Affiliation of author	
Date of publication *(year, month, date)*	

Characteristics of included studies

Methods

	Descriptions as stated in report/paper	Location in text or source *(pg & ¶/fig/table/other)*
Country		
City		
Data collected from date *(year, month, date)*		
Data collected until the date *(year, month, date)*		
Method used to calculate reproduction number		
Model used to calculate reproduction number		
Assumption of the model		
Sample size		
Notes:		

Outcome: Reproduction number

	Description	Location in text or source *(pg & ¶/fig/table/other)*
Type of reproduction number assessed		
Reproduction number		
Interval of reproduction number		
SD or SE *(if mentioned)*		
Type of interval *(95% CI, 90% CI, max/min, Credible Interval)*		
Type of central estimate *(mean, median)*		
Notes:		

Study quality assessment through National Institute of Health (NIH) quality assessment tool for case series studies

Criteria	Yes	No	Other (CD, NR, NA)*
1. Was the study question or objective clearly stated?	☐	☐	
2. Was the study population clearly and fully described, including a case definition?	☐	☐	
3. Were the cases consecutive?	☐	☐	
4. Were the subjects comparable?	☐	☐	
5. Was the intervention clearly described?	☐	☐	
6. Were the outcome measures clearly defined, valid, reliable, and implemented consistently across all study participants?	☐	☐	
7. Was the length of follow-up adequate?	☐	☐	
8. Were the statistical methods well-described?	☐	☐	
9. Were the results well-described?	☐	☐	

Quality Rating (Good, Fair, or Poor)
Additional Comments (If POOR, please state why):

*CD, cannot determine; NA, not applicable; NR, not reported
