# Supplementary material for: Reliability of Early Estimates of the Basic Reproduction Number of COVID-19: A Systematic Review and Meta-Analysis
Source: Int J Environ Res Public Health. 2022 Sep 15;19(18):11613. doi: 10.3390/ijerph191811613 (PMC9517346; doi:10.3390/ijerph191811613)
Supplement: Supplementary file 1 [file ijerph-19-11613-s001.zip › Supplementary file S5.pdf]

References of the 151 articles included in the systematic review

1. Xue L, Jing S, Miller JC, et al. A data-driven network model for the emerging COVID-19 epidemics in Wuhan, Toronto and Italy. *Mathematical Biosciences*. 2020;326.
2. Iwata K, Miyakoshi C. A Simulation on Potential Secondary Spread of Novel Coronavirus in an Exported Country Using a Stochastic Epidemic SEIR Model. *Journal of Clinical Medicine*. 2020;9(4):944.
3. Kassa SM, Njagarah JBH, Terefe YA. Analysis of the mitigation strategies for COVID-19: From mathematical modelling perspective. *Chaos, Solitons & Fractals*. 2020;138:109968.
4. Wang X, Pan Y, Zhang D, et al. Basic epidemiological parameter values from data of real-world in mega-cities: The characteristics of COVID-19 in Beijing, China. *BMC Infectious Diseases*. 2020;20(1).
5. Lau MSY, Grenfell B, Thomas M, Bryan M, Nelson K, Lopman B. Characterizing superspreading events and age-specific infectiousness of SARS-CoV-2 transmission in Georgia, USA. *Proceedings of the National Academy of Sciences of the United States of America*. 2020;117(36):22430-22435.
6. Biswas SK, Ghosh JK, Sarkar S, Ghosh U. COVID-19 pandemic in India: a mathematical model study. *Nonlinear Dynamics*. 2020;102(1):537-553.
7. Dropkin G. COVID-19 UK Lockdown Forecasts and R0. *Frontiers in Public Health*. 2020;8.
8. Wang K, Lu Z, Wang X, et al. Current trends and future prediction of novel coronavirus disease (COVID-19) epidemic in China: a dynamical modeling analysis. *Mathematical Biosciences and Engineering*. 2020;17(4):3052-3061.
9. Anastassopoulou C, Russo L, Tsakris A, Siettos C. Data-based analysis, modelling and forecasting of the COVID-19 outbreak. *PLoS ONE*. 2020;15(3).
10. Bhaskar A, Ponnuraja C, Srinivasan R, Padmanaban S. Distribution and growth rate of COVID-19 outbreak in Tamil Nadu: A log-linear regression approach. *Indian journal of public health*. 2020;64(Supplement):S188-S191.
11. Mizumoto K, Kagaya K, Chowell G. Early epidemiological assessment of the transmission potential and virulence of coronavirus disease 2019 (COVID-19) in Wuhan City, China, January–February, 2020. *BMC Medicine*. 2020;18(1):217.

12. Li Q, Guan X, Wu P, et al. Early transmission dynamics in Wuhan, China, of novel coronavirus-infected pneumonia. *New England Journal of Medicine*. 2020;382(13):1199-1207.
13. Munayco C v, Tariq A, Rothenberg R, et al. Early transmission dynamics of COVID-19 in a southern hemisphere setting: Lima-Peru: February 29th–March 30th, 2020. *Infectious Disease Modelling*. 2020;5:338-345.
14. Ki M. Epidemiologic characteristics of early cases with 2019 novel coronavirus (2019-nCoV) disease in Korea. *Epidemiology and health*. 2020;42:e2020007.
15. Haw NJL, Uy J, Sy KTL, Abrigo MRM. Epidemiological profile and transmission dynamics of COVID-19 in the Philippines. *Epidemiology and infection*. 2020;148:e204.
16. Bi Q, Wu Y, Mei S, et al. Epidemiology and transmission of COVID-19 in 391 cases and 1286 of their close contacts in Shenzhen, China: a retrospective cohort study. *The Lancet Infectious Diseases*. 2020;20(8):911-919.
17. Lonergan M, Chalmers JD. Estimates of the ongoing need for social distancing and control measures post-“lockdown” from trajectories of COVID-19 cases and mortality. *European Respiratory Journal*. 2020;56(1).
18. Zhao S, Musa SS, Lin Q, et al. Estimating the unreported number of novel coronavirus (2019-ncov) cases in China in the first half of january 2020: A data-driven modelling analysis of the early outbreak. *Journal of Clinical Medicine*. 2020;9(2).
19. Musa SS, Zhao S, Wang MH, Habib AG, Mustapha UT, He D. Estimation of exponential growth rate and basic reproduction number of the coronavirus disease 2019 (COVID-19) in Africa. *Infect Dis Poverty*. 2020;9(1):96.
20. Chae SY, Lee K, Lee HM, et al. Estimation of Infection Rate and Predictions of Disease Spreading Based on Initial Individuals Infected With COVID-19. *Frontiers in Physics*. 2020;8.
21. Xu C, Dong Y, Yu X, et al. Estimation of reproduction numbers of COVID-19 in typical countries and epidemic trends under different prevention and control scenarios. *Frontiers of Medicine*. 2020;14(5):613-622.
22. PANG L, LIU S, ZHANG X, TIAN T, ZHAO Z. Transmission dynamics and control strategies of covid-19 in Wuhan, China. *Journal of Biological Systems*. 2020;28(03):543-560.

23. Hoque ME. Estimation of the number of affected people due to the Covid-19 pandemic using susceptible, infected and recover model. *International Journal of Modern Physics C*. 2020;31(08):2050111.
24. Kanagarathinam K, Sekar K. Estimation of the reproduction number and early prediction of the COVID-19 outbreak in India using a statistical computing approach. *Epidemiology and health*. 2020;42:e2020028.
25. Zhang S, Diao M, Yu W, Pei L, Lin Z, Chen D. Estimation of the reproductive number of novel coronavirus (COVID-19) and the probable outbreak size on the Diamond Princess cruise ship: A data-driven analysis. *International Journal of Infectious Diseases*. 2020;93:201-204.
26. You C, Deng Y, Hu W, et al. Estimation of the time-varying reproduction number of COVID-19 outbreak in China. *International journal of hygiene and environmental health*. 2020;228:113555.
27. Maugeri A, Barchitta M, Battiato S, Agodi A. Estimation of unreported novel coronavirus (Sars-coV-2) infections from reported deaths: A susceptible–exposed–infectious–recovered–dead model. *Journal of Clinical Medicine*. 2020;9(5).
28. Glass DH. European and US lockdowns and second waves during the COVID-19 pandemic. *Mathematical Biosciences*. 2020;330.
29. Zhang Y, Li Y, Wang L, Li M, Zhou X. Evaluating transmission heterogeneity and super-spreading event of COVID-19 in a metropolis of China. *International Journal of Environmental Research and Public Health*. 2020;17(10).
30. Kim S, Jeong YD, Byun JH, et al. Evaluation of COVID-19 epidemic outbreak caused by temporal contact-increase in South Korea. *International Journal of Infectious Diseases*. 2020;96:454-457.
31. Su L, Hong N, Zhou X, et al. Evaluation of the Secondary Transmission Pattern and Epidemic Prediction of COVID-19 in the Four Metropolitan Areas of China. *Frontiers in Medicine*. 2020;7.
32. Grzybowski JM v., da Silva R v., Rafikov M. Expanded SEIRCQ Model Applied to COVID-19 Epidemic Control Strategy Design and Medical Infrastructure Planning. *Mathematical Problems in Engineering*. 2020;2020:1-15.
33. Wangping J, Ke H, Yang S, et al. Extended SIR Prediction of the Epidemics Trend of COVID-19 in Italy and Compared With Hunan, China. *Frontiers in Medicine*. 2020;7.

34. Chen Z, Yang J, Dai B. Forecast possible risk for COVID-19 epidemic dissemination under current control strategies in Japan. *International Journal of Environmental Research and Public Health*. 2020;17(11).

35. Wirawan A, Januraga PP. Forecasting COVID-19 Transmission and Healthcare Capacity in Bali, Indonesia. *Journal of Preventive Medicine and Public Health*. 2020;53(3):158-163.

36. Khajanchi S, Sarkar K. Forecasting the daily and cumulative number of cases for the COVID-19 pandemic in India. *Chaos: An Interdisciplinary Journal of Nonlinear Science*. 2020;30(7):071101.

37. Almeshal AM, Almazrouee AI, Alenizi MR, Alhajeri SN. Forecasting the Spread of COVID-19 in Kuwait Using Compartmental and Logistic Regression Models. *Applied Sciences*. 2020;10(10):3402.

38. Aviv-Sharon E, Aharoni A. Generalized logistic growth modeling of the COVID-19 pandemic in Asia. *Infectious Disease Modelling*. 2020;5:502-509.

39. Katul GG, Mrad A, Bonetti S, Manoli G, Parolari AJ. Global convergence of COVID-19 basic reproduction number and estimation from early-time SIR dynamics. *PLoS ONE*. 2020;15(9 September).

40. Zhao S, Stone L, Gao D, et al. Imitation dynamics in the mitigation of the novel coronavirus disease (COVID-19) outbreak in Wuhan, China from 2019 to 2020. *Annals of Translational Medicine*. 2020;8(7).

41. Yang J, Wang G, Zhang S, Xu F, Li X. Analysis of the age-structured epidemiological characteristics of SARS-COV-2 transmission in mainland China: An aggregated approach. Augeraud E, Banerjee M, Dhersin JS, et al., eds. *Mathematical Modelling of Natural Phenomena*. 2020;15:39.

42. Chaves LF, Hurtado LA, Rojas MR, Friberg MD, Rodríguez RM, Avila-Aguero ML. COVID-19 basic reproduction number and assessment of initial suppression policies in Costa Rica. Augeraud E, Banerjee M, Dhersin JS, et al., eds. *Mathematical Modelling of Natural Phenomena*. 2020;15:32.

43. Salje H, Kiem CT, Lefrancq N, et al. Estimating the burden of SARS-CoV-2 in France. *Science*. 2020;369(6500):208-211.

44. Xu C, Yu Y, Chen Y, Lu Z. Forecast analysis of the epidemics trend of COVID-19 in the USA by a generalized fractional-order SEIR model. *Nonlinear Dynamics*. 2020;101(3):1621-1634.

45. di Domenico L, Pullano G, Sabbatini CE, Boëlle PY, Colizza V. Impact of lockdown on COVID-19 epidemic in Île-de-France and possible exit strategies. *BMC Medicine*. 2020;18(1).
46. Patel P, Athotra A, Vaisakh TP, Dikid T, Jain SK. Impact of nonpharmacological interventions on COVID-19 transmission dynamics in India. *Indian journal of public health*. 2020;64(Supplement):S142-S146.
47. Wang K, Zhao S, Liao Y, et al. Estimating the serial interval of the novel coronavirus disease (COVID-19) based on the public surveillance data in Shenzhen, China, from 19 January to 22 February 2020. *Transboundary and Emerging Diseases*. Published online 2020.
48. Sanche S, Lin YT, Xu C, Romero-Severson E, Hengartner N, Ke R. High Contagiousness and Rapid Spread of Severe Acute Respiratory Syndrome Coronavirus 2. *Emerging Infectious Diseases*. 2020;26(7):1470-1477.
49. Patrikar SR, Kotwal A, Bhatti VK, et al. Incubation Period and Reproduction Number for Novel Coronavirus 2019 (COVID-19) Infections in India. *Asia Pacific Journal of Public Health*. 2020;32(8):458-460.
50. Kwok KO, Chan HHH, Huang Y, et al. Inferring super-spreading from transmission clusters of COVID-19 in Hong Kong, Japan, and Singapore. *Journal of Hospital Infection*. 2020;105(4):682-685.
51. Willis MJ, Díaz VHG, Prado-Rubio OA, von Stosch M. Insights into the dynamics and control of COVID-19 infection rates. *Chaos, Solitons & Fractals*. 2020;138:109937.
52. Adekunle AI, Adegboye OA, Gayawan E, McBryde ES. Is Nigeria really on top of COVID-19? Message from effective reproduction number. *Epidemiology and Infection*. Published online 2020.
53. Bae TW, Kwon KK, Kim KH. Mass Infection Analysis of COVID-19 Using the SEIRD Model in Daegu-Gyeongbuk of Korea from April to May, 2020. *Journal of Korean medical science*. 2020;35(34):e317.
54. Naveed M, Rafiq M, Raza A, et al. Mathematical Analysis of Novel Coronavirus (2019-nCov) Delay Pandemic Model. *Computers, Materials & Continua*. 2020;64(3):1401-1414.
55. Yang HM, Lombardi Junior LP, Castro FFM, Yang AC. Mathematical model describing CoViD-19 in São Paulo, Brazil - evaluating isolation as control mechanism and forecasting epidemiological scenarios of release. *Epidemiology and infection*. 2020;148:e155.

56. Veera Krishna M. Mathematical modelling on diffusion and control of COVID–19. *Infectious Disease Modelling*. 2020;5:588-597.
57. Jiang S, Li Q, Li C, et al. Mathematical models for devising the optimal SARS-CoV-2 strategy for eradication in China, South Korea, and Italy. *Journal of Translational Medicine*. 2020;18(1).
58. Tian J, Wu J, Bao Y, et al. Modeling analysis of COVID-19 based on morbidity data in Anhui, China. *Mathematical Biosciences and Engineering*. 2020;17(4):2842-2852.
59. Irvine M, Coombs D, Skarha J, et al. Modeling COVID-19 and Its Impacts on U.S. Immigration and Customs Enforcement (ICE) Detention Facilities, 2020. *Journal of urban health : bulletin of the New York Academy of Medicine*. 2020;97(4):439-447.
60. Turk PJ, Chou SH, Kowalkowski MA, et al. Modeling COVID-19 Latent Prevalence to Assess a Public Health Intervention at a State and Regional Scale: Retrospective Cohort Study. *JMIR public health and surveillance*. 2020;6(2):e19353.
61. Saldaña F, Flores-Arguedas H, Camacho-Gutiérrez JA, Barradas I. Modeling the transmission dynamics and the impact of the control interventions for the COVID-19 epidemic outbreak. *Mathematical Biosciences and Engineering*. 2020;17(4):4165-4183.
62. Wang L, Wang J, Zhao H, et al. Modelling and assessing the effects of medical resources on transmission of novel coronavirus (COVID-19) in Wuhan, China. *Mathematical Biosciences and Engineering*. 2020;17(4):2936-2949.
63. Marimuthu S, Joy M, Malavika B, et al. Modelling of reproduction number for COVID-19 in India and high incidence states. *Clinical Epidemiology and Global Health*. Published online 2020.
64. Goscé L, Phillips PA, Spinola P, Gupta DRK, Abubakar PI. Modelling SARS-COV2 Spread in London: Approaches to Lift the Lockdown. *Journal of Infection*. 2020;81(2):260-265.
65. Feng LX, Jing SL, Hu SK, Wang DF, Huo HF. Modelling the effects of media coverage and quarantine on the COVID-19 infections in the UK. *Mathematical Biosciences and Engineering*. 2020;17(4):3618-3636.
66. Wu JT, Leung K, Leung GM. Nowcasting and forecasting the potential domestic and international spread of the 2019-nCoV outbreak originating in Wuhan, China: a modelling study. *The Lancet*. 2020;395(10225):689-697.
67. Zhu Y, Chen YQ. On a Statistical Transmission Model in Analysis of the Early Phase of COVID-19 Outbreak. *Statistics in Biosciences*. Published online 2020.

68. Peirlinck M, Linka K, Sahli Costabal F, Kuhl E. Outbreak dynamics of COVID-19 in China and the United States. *Biomechanics and Modeling in Mechanobiology*. Published online 2020.
69. Linka K, Peirlinck M, Sahli Costabal F, Kuhl E. Outbreak dynamics of COVID-19 in Europe and the effect of travel restrictions. *Computer Methods in Biomechanics and Biomedical Engineering*. 2020;23(11):710-717.
70. Bentout S, Chekroun A, Kuniya T. Parameter estimation and prediction for coronavirus disease outbreak 2019 (COVID-19) in Algeria. *AIMS public health*. Published online 2020.
71. Riou J, Althaus CL. Pattern of early human-to-human transmission of Wuhan 2019 novel coronavirus (2019-nCoV), December 2019 to January 2020. *Eurosurveillance*. 2020;25(4).
72. Feng X, Chen J, Wang K, et al. Phase-adjusted estimation of the COVID-19 outbreak in South Korea under multi-source data and adjustment measures: a modelling study. *Mathematical Biosciences and Engineering*. 2020;17(4):3637-3648.
73. Taboe HB, Salako K v, Tison JM, Ngonghala CN, Glèlè Kakaï R. Predicting COVID-19 spread in the face of control measures in West Africa. *Mathematical Biosciences*. 2020;328.
74. Liu Z, Magal P, Seydi O, Webb G. Predicting the cumulative number of cases for the COVID-19 epidemic in China from early data. *Mathematical Biosciences and Engineering*. 2020;17(4):3040-3051.
75. Moussaoui A, Auger P. Prediction of confinement effects on the number of Covid-19 outbreak in Algeria. Augeraud E, Banerjee M, Dhersin JS, et al., eds. *Mathematical Modelling of Natural Phenomena*. 2020;15:37.
76. Alsayed A, Sadir H, Kamil R, Sari H. Prediction of epidemic peak and infected cases for COVID-19 disease in Malaysia, 2020. *International Journal of Environmental Research and Public Health*. 2020;17(11):1-15.
77. Kuniya T. Prediction of the Epidemic Peak of Coronavirus Disease in Japan, 2020. *Journal of Clinical Medicine*. 2020;9(3):789.
78. Zhuang Z, Zhao S, Lin Q, et al. Preliminary estimates of the reproduction number of the coronavirus disease (COVID-19) outbreak in Republic of Korea and Italy by 5 March 2020. *International Journal of Infectious Diseases*. 2020;95:308-310.

79. Rotejanaprasert C, Lawpoolsri S, Pan-Ngum W, Maude RJ. Preliminary estimation of temporal and spatiotemporal dynamic measures of COVID- 19 transmission in Thailand. *PLoS ONE*. 2020;15(9 September).
80. Zhao S, Lin Q, Ran J, et al. Preliminary estimation of the basic reproduction number of novel coronavirus (2019-nCoV) in China, from 2019 to 2020: A data-driven analysis in the early phase of the outbreak. *International Journal of Infectious Diseases*. 2020;92:214-217.
81. Zhou T, Liu Q, Yang Z, et al. Preliminary prediction of the basic reproduction number of the Wuhan novel coronavirus 2019-nCoV. *Journal of Evidence-Based Medicine*. 2020;13(1):3-7.
82. Wang K, Zhao S, Li H, et al. Real-time estimation of the reproduction number of the novel coronavirus disease (COVID-19) in China in 2020 based on incidence data. *Annals of translational medicine*. Published online 2020.
83. Jung SM, Akhmetzhanov AR, Hayashi K, et al. Real-time estimation of the risk of death from novel coronavirus (COVID-19) infection: Inference using exported cases. *Journal of Clinical Medicine*. 2020;9(2).
84. Hao X, Cheng S, Wu D, Wu T, Lin X, Wang C. Reconstruction of the full transmission dynamics of COVID-19 in Wuhan. *Nature*. 2020;584(7821):420-424.
85. Buckman SR, Glick R, Lansing KJ, Petrosky-Nadeau N, Seitelman LM. Replicating and projecting the path of COVID-19 with a model-implied reproduction number. *Infectious Disease Modelling*. 2020;5:635-651.
86. Kim S, Choi S, Ko Y, Ki M, Jung E. Risk estimation of the SARS-CoV-2 acute respiratory disease outbreak outside China. *Theoretical biology & medical modelling*. 2020;17(1):9.
87. Nguemdjo U, Meno F, Dongfack A, Ventelou B. Simulating the progression of the COVID-19 disease in Cameroon using SIR models. *PLoS ONE*. 2020;15(8 August 2020).
88. Huang R, Liu M, Ding Y. Spatial-temporal distribution of COVID-19 in China and its prediction: A data-driven modeling analysis. *Journal of Infection in Developing Countries*. 2020;14(3):246-253.
89. Gatto M, Bertuzzo E, Mari L, et al. Spread and dynamics of the COVID-19 epidemic in Italy: Effects of emergency containment measures. *Proceedings of the National Academy of Sciences of the United States of America*. 2020;117(19):10484-10491.

90. Khosravi A, Chaman R, Rohani-Rasaf M, Zare F, Mehravaran S, Emamian MH. The basic reproduction number and prediction of the epidemic size of the novel coronavirus (COVID-19) in Shahroud, Iran. *Epidemiology and Infection*. Published online 2020.
91. Al-Raeei M. The basic reproduction number of the new coronavirus pandemic with mortality for India, the Syrian Arab Republic, the United States, Yemen, China, France, Nigeria and Russia with different rate of cases. *Clinical Epidemiology and Global Health*. Published online 2020.
92. Guirao A. The Covid-19 outbreak in Spain. A simple dynamics model, some lessons, and a theoretical framework for control response. *Infectious Disease Modelling*. 2020;5:652-669.
93. el Deeb O, Jalloul M. The dynamics of COVID-19 spread: evidence from Lebanon. *Mathematical Biosciences and Engineering*. 2020;17(5):5618-5632.
94. Khan MA, Atangana A, Alzahrani E, Fatmawati. The dynamics of COVID-19 with quarantined and isolation. *Advances in Difference Equations*. 2020;2020(1):425.
95. Linka K, Peirlinck M, Kuhl E. The reproduction number of COVID-19 and its correlation with public health interventions. *Computational Mechanics*. 2020;66(4):1035-1050.
96. Ali M, Shah STH, Imran M, Khan A. The role of asymptomatic class, quarantine and isolation in the transmission of COVID-19. *Journal of Biological Dynamics*. 2020;14(1):389-408.
97. Li C, Xu J, Liu J, Zhou Y. The within-host viral kinetics of SARS-CoV-2. *Mathematical Biosciences and Engineering*. 2020;17(4):2853-2861.
98. Dur-e-Ahmad M, Imran M. Transmission Dynamics Model of Coronavirus COVID-19 for the Outbreak in Most Affected Countries of the World. *International Journal of Interactive Multimedia and Artificial Intelligence*. 2020;6(2):4.
99. Sharov KS. Trends in adaptation of fifteen European countries population to SARS-CoV-2 in March–May 2020: Can Taiwanese experience be adopted? *Journal of the Formosan Medical Association*. Published online 2020.
100. Mena RH, Velasco-Hernandez JX, Mantilla-Beniers NB, et al. Using posterior predictive distributions to analyse epidemic models: COVID-19 in Mexico City. *Physical biology*. 2020;17(6):65001.

101. Liu F, Li X, Zhu G. Using the contact network model and Metropolis-Hastings sampling to reconstruct the COVID-19 spread on the "Diamond Princess." *Science Bulletin*. 2020;65(15):1297-1305.
102. Song H, Li F, Jia Z, Jin Z, Liu S. Using traveller-derived cases in Henan Province to quantify the spread of COVID-19 in Wuhan, China. *Nonlinear Dynamics*. 2020;101(3):1821-1831.
103. Wan K, Chen J, Lu C, Dong L, Wu Z, Zhang L. When will the battle against novel coronavirus end in Wuhan: A SEIR modeling analysis. *Journal of global health*. 2020;10(1):11002.
104. Li J. A Robust Stochastic Method of Estimating the Transmission Potential of 2019-nCoV. *arXiv*. Published online February 7, 2020.
105. Zhao Q, Chen Y, Small DS. Analysis of the epidemic growth of the early 2019-nCoV outbreak using internationally confirmed cases. *medRxiv*. Published online February 9, 2020.
106. Zhou H, Xue C, Gao G, Lawless L, Xie L, Zhang KK. Characterizing the transmission and identifying the control strategy for COVID-19 through epidemiological modeling. *medRxiv*. Published online February 25, 2020.
107. Bai Y, Nie X, Wen C. Epidemic Prediction of 2019-nCoV in Hubei Province and Comparison with SARS in Guangdong Province. *SSRN pre print*.
108. Cao Z, Zhang Q, Lu X, et al. Estimating the effective reproduction number of the 2019-nCoV in China. *medRxiv*. Published online January 29, 2020.
109. Li J, Wang Y, Gilmour S, et al. Estimation of the epidemic properties of the 2019 novel coronavirus: A mathematical modeling study. *medRxiv*. Published online February 20, 2020.
110. Tang B, Wang X, Li Q, et al. Estimation of the Transmission Risk of the 2019-nCoV and Its Implication for Public Health Interventions. *Journal of Clinical Medicine*. 2020;9(2).
111. Zhou C. Evaluating new evidence in the early dynamics of the novel coronavirus COVID-19 outbreak in Wuhan, China with real time domestic traffic and potential asymptomatic transmissions. *medRxiv*. Published online February 20, 2020.
112. Cao Z, Zhang Q, Lu X, et al. Incorporating Human Movement Data to Improve Epidemiological Estimates for 2019-nCoV. *medRxiv*. Published online February 9, 2020.

113. Ivorra B, Ferrández MR, Vela-Pérez M, Ramos AM. Mathematical modeling of the spread of the coronavirus disease 2019 (COVID-19) taking into account the undetected infections. The case of China. *Communications in Nonlinear Science & Numerical Simulation*. 2020;88:105303.
114. Shen M, Peng Z, Xiao Y, Zhang L. Modelling the epidemic trend of the 2019 novel coronavirus outbreak in China. *bioRxiv*. Published online January 25, 2020.
115. Read JM, Bridgen JRE, Cummings DAT, Ho A, Jewell CP. Novel coronavirus 2019-nCoV: early estimation of epidemiological parameters and epidemic predictions. *medRxiv*. Published online January 28, 2020.
116. Imai N, Cori A, Dorigatti I, et al. *Report 3: Transmissibility of 2019-NCoV.*; 2020.
117. Xiong H, Yan H. Simulating the Infected Population and Spread Trend of 2019-nCov Under Different Policy by EIR Model. *SSRN preprint*. Published online February 8, 2020.
118. Li R, Pei S, Chen B, et al. Substantial undocumented infection facilitates the rapid dissemination of novel coronavirus (SARS-CoV-2). *Science*. 2020;368(6490):489-493.
119. Sanche S, Lin YT, Xu C, Romero-Severson E, Hengartner N, Ke R. The Novel Coronavirus, 2019-nCoV, is Highly Contagious and More Infectious Than Initially Estimated [Preprint]. *medRxiv*. Published online February 11, 2020.
120. Shao N, Cheng J, Chen W. The reproductive number R0 of COVID-19 based on estimate of a statistical time delay dynamical system [Preprint]. *medRxiv*. Published online February 20, 2020.
121. Abbott S, Hellewell J, Munday J, Funk S. The transmissibility of novel Coronavirus in the early stages of the 2019-20 outbreak in Wuhan: Exploring initial point-source exposure sizes and durations using scenario analysis. *Wellcome Open Research 2020 5:17*. 2020;5:17.
122. Paiva HM, Afonso RJM, de Oliveira IL, Garcia GF. A data-driven model to describe and forecast the dynamics of COVID-19 transmission. *PLoS ONE*. 2020;15(7 July).
123. He S, Tang S, Rong L. A discrete stochastic model of the COVID-19 outbreak: Forecast and control. *Mathematical Biosciences and Engineering*. 2020;17(4):2792-2804.
124. Lu Z, Yu Y, Chen Y, et al. A fractional-order SEIHDR model for COVID-19 with inter-city networked coupling effects. *Nonlinear Dynamics*. 2020;101(3):1717-1730.

125. Chen TM, Rui J, Wang QP, Zhao ZY, Cui JA, Yin L. A mathematical model for simulating the phase-based transmissibility of a novel coronavirus. *Infectious Diseases of Poverty*. 2020;9(1).
126. Yang CY, Wang J. A mathematical model for the novel coronavirus epidemic in Wuhan, China. *Mathematical biosciences and engineering : MBE*. 2020;17(3):2708-2724.
127. Shaikh AS, Shaikh IN, Nisar KS. A mathematical model of COVID-19 using fractional derivative: outbreak in India with dynamics of transmission and control. *Advances in Difference Equations*. 2020;2020(1):373.
128. Zongo P, Zorom M, Mophou G, Dorville R, Beaumont C. A model of COVID-19 transmission to understand the effectiveness of the containment measures: application to data from France. *Epidemiology and infection*. 2020;148:e221.
129. Mandal M, Jana S, Nandi SK, Khatua A, Adak S, Kar TK. A model based study on the dynamics of COVID-19: Prediction and control. *Chaos, Solitons & Fractals*. 2020;136:109889.
130. Carcione JM, Santos JE, Bagaini C, Ba J. A Simulation of a COVID-19 Epidemic Based on a Deterministic SEIR Model. *Frontiers in Public Health*. 2020;8.
131. ben Hassen H, Elaoud A, ben Salah N, Masmoudi A. A SIR-Poisson Model for COVID-19: Evolution and Transmission Inference in the Maghreb Central Regions. *Arabian Journal for Science and Engineering*. 2021;46(1):93-102.
132. Ali M, Imran M, Khan A. Analysis and prediction of the COVID-19 outbreak in Pakistan. *Journal of Biological Dynamics*. 2020;14(1):730-747.
133. Belgaid Y, Helal M, Venturino E. Analysis of a Model for Coronavirus Spread. *Mathematics*. 2020;8(5):820.
134. Fitzgibbon WE, Morgan JJ, Webb GF, Wu Y. Analysis of a reaction–diffusion epidemic model with asymptomatic transmission. *Journal of Biological Systems*. 2020;28(03):561-587.
135. Li Y, Wang LW, Peng ZH, Shen HB. Basic reproduction number and predicted trends of coronavirus disease 2019 epidemic in the mainland of China. *Infectious Diseases of Poverty*. 2020;9(1):94.
136. Madubueze CE, Dachollom S, Onwubuya IO. Controlling the Spread of COVID-19: Optimal Control Analysis. *Computational and mathematical methods in medicine*. 2020;2020:6862516.

137. Lakman IA, Agapitov AA, Sadikova LF, et al. COVID-19 mathematical forecasting in the Russian Federation. *Arterial'naya Gipertenziya (Arterial Hypertension)*. 2020;26(3):288-294.
138. Djilali S, Ghanbari B. Coronavirus pandemic: A predictive analysis of the peak outbreak epidemic in South Africa, Turkey, and Brazil. *Chaos, Solitons & Fractals*. 2020;138:109971.
139. Rocklöv J, Sjödin H, Wilder-Smith A. COVID-19 outbreak on the Diamond Princess cruise ship: estimating the epidemic potential and effectiveness of public health countermeasures. *Journal of Travel Medicine*. 2020;27(3).
140. Distante C, Piscitelli P, Miani A. Covid-19 outbreak progression in italian regions: Approaching the peak by the end of march in northern Italy and first week of april in southern Italy. *International Journal of Environmental Research and Public Health*. 2020;17(9).
141. Rong X, Yang L, Chu H, Fan M. Effect of delay in diagnosis on transmission of COVID-19. *Mathematical Biosciences and Engineering*. 2020;17(3):2725-2740.
142. Radwan GN. Epidemiology of sars-cov-2 in Egypt. *Eastern Mediterranean Health Journal*. 2020;26(7):768-773.
143. Hilton J, Keeling MJ. Estimation of country-level basic reproductive ratios for novel coronavirus (sars-cov-2/ covid-19) using synthetic contact matrices. *PLoS Computational Biology*. 2020;16(7).
144. Bryant P, Elofsson A. Estimating the impact of mobility patterns on COVID-19 infection rates in 11 European countries. *PeerJ*. 2020;8:e9879.
145. Liu M, Ning J, Du Y, et al. Modelling the evolution trajectory of COVID-19 in Wuhan, China: experience and suggestions. *Public Health*. 2020;183:76.
146. Loli Piccolomini E, Zama F, Piccolomini EL, Zama F. Monitoring Italian COVID-19 spread by a forced SEIRD model. *PloS one*. 2020;15(8 August):e0237417.
147. Fredj H ben, Chérif F. Novel Corona virus disease infection in Tunisia: Mathematical model and the impact of the quarantine strategy. *Chaos, Solitons & Fractals*. 2020;138:109969.
148. Blasius B. Power-law distribution in the number of confirmed COVID-19 cases. *Chaos: An Interdisciplinary Journal of Nonlinear Science*. 2020;30(9):093123.
149. Khan MA, Atangana A. Modeling the dynamics of novel coronavirus (2019-nCov) with fractional derivative. *Alexandria Engineering Journal*. 2020;59(4):2379-2389.

150. Huang Q, Kang YS. Mathematical Modeling of COVID-19 Control and Prevention Based on Immigration Population Data in China: Model Development and Validation. *JMIR public health and surveillance*. 2020;6(2):e18638.

151. al Zobbi M, Alsinglawi B, Mubin O, Alnajjar F. Measurement Method for Evaluating the Lockdown Policies during the COVID-19 Pandemic. *International journal of environmental research and public health*. 2020;17(15).
